# Supplementary material for: In vivo evaluation of histopathologic findings of vascular damage after mechanical thrombectomy with the Tromba device in a canine model of cerebral infarction
Source: PLoS One. 2022 Oct 14;17(10):e0276108. doi: 10.1371/journal.pone.0276108 (PMC9565453; doi:10.1371/journal.pone.0276108)

Raw histopathology images

No.1 H&E (50X)

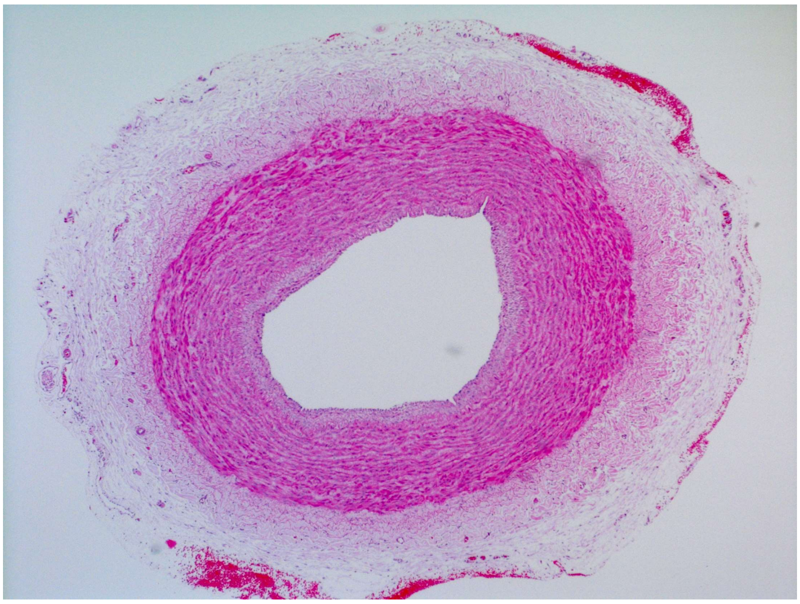

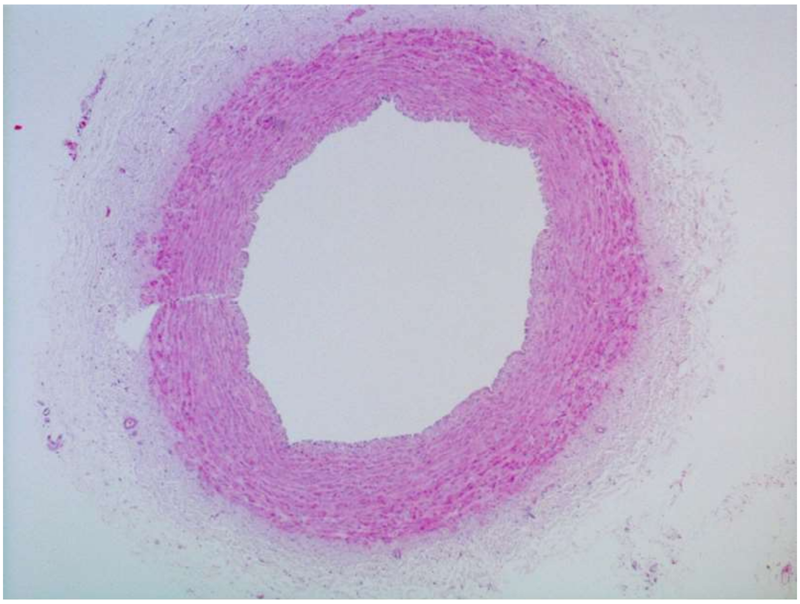

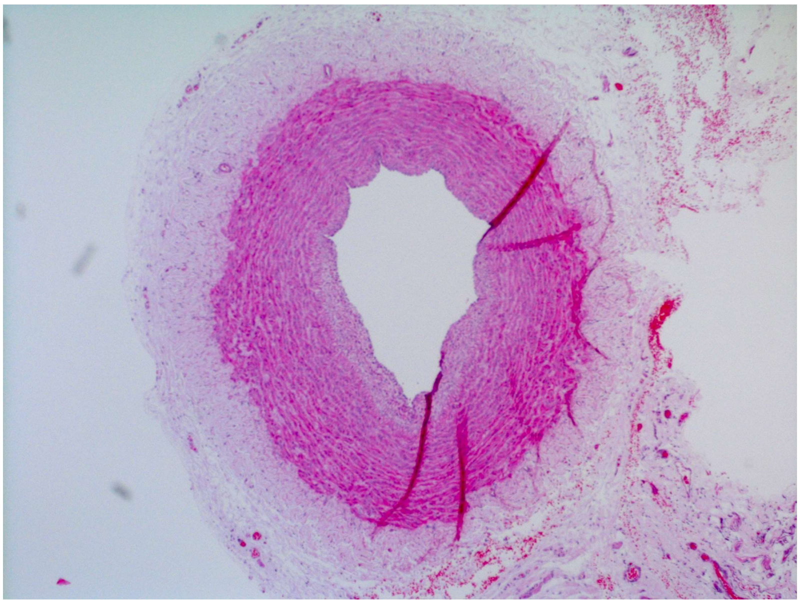

No.1 Masson's Trichrome (50X)

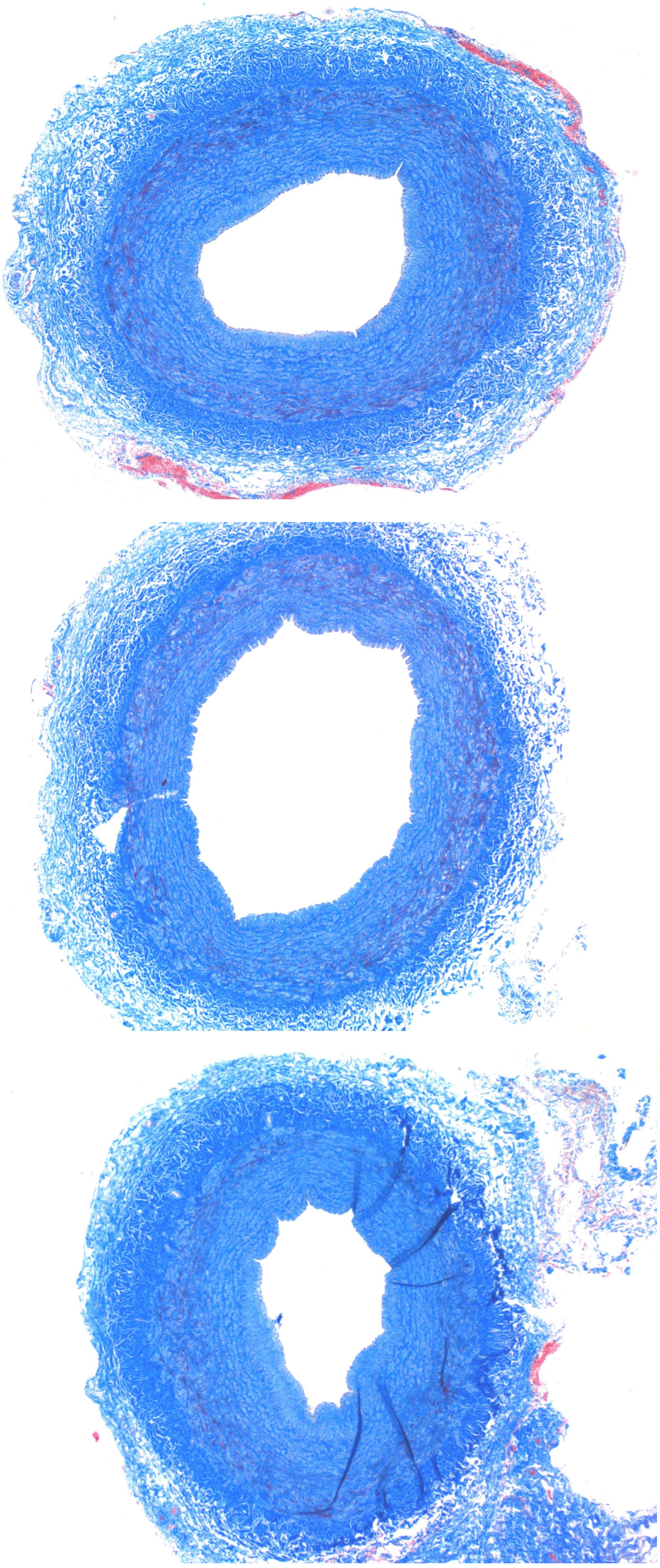

No.2 H&E (50X)

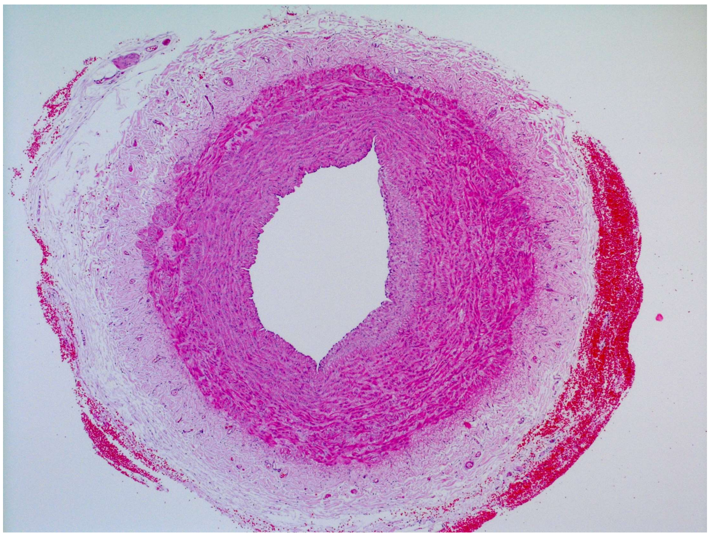

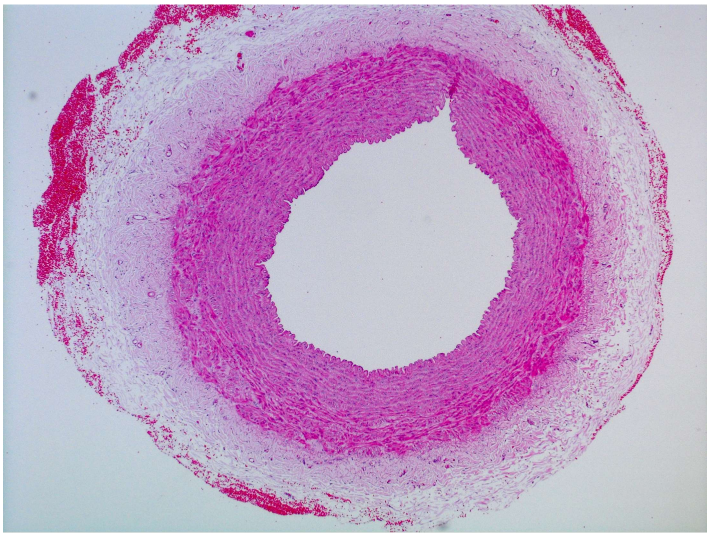

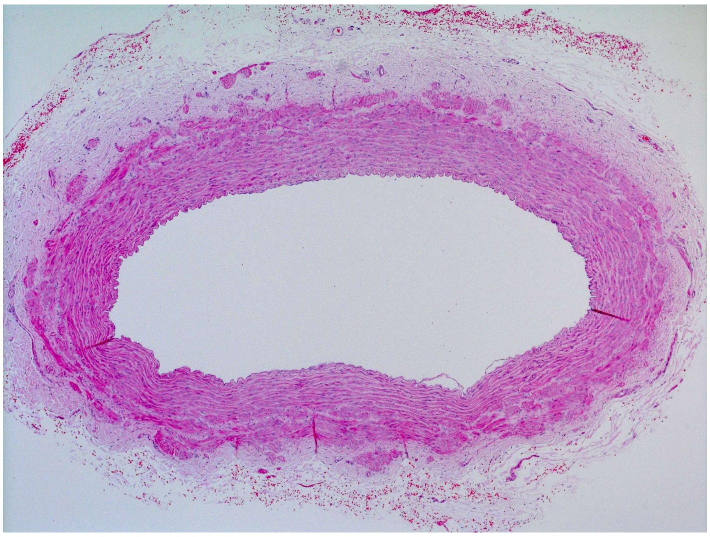

No.2 Masson's Trichrome (50X)

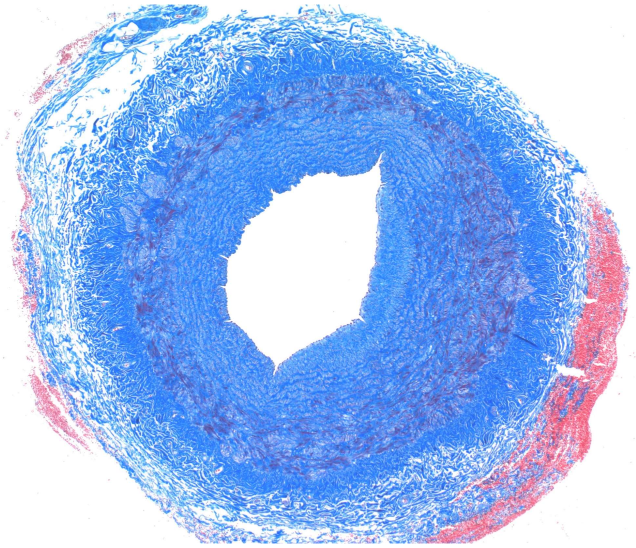

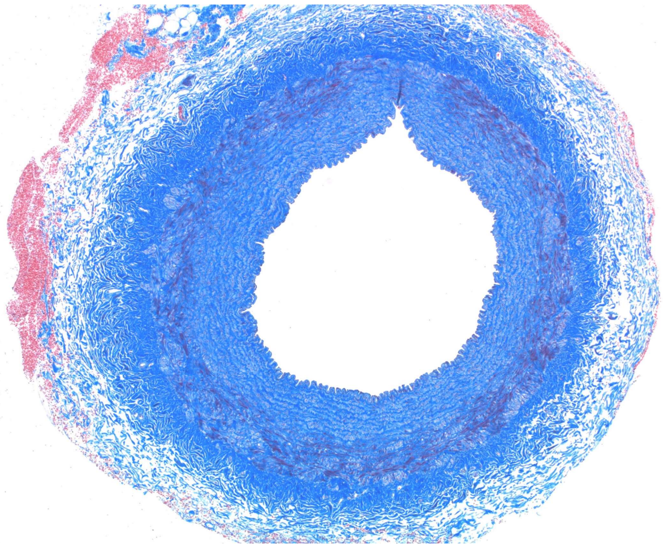

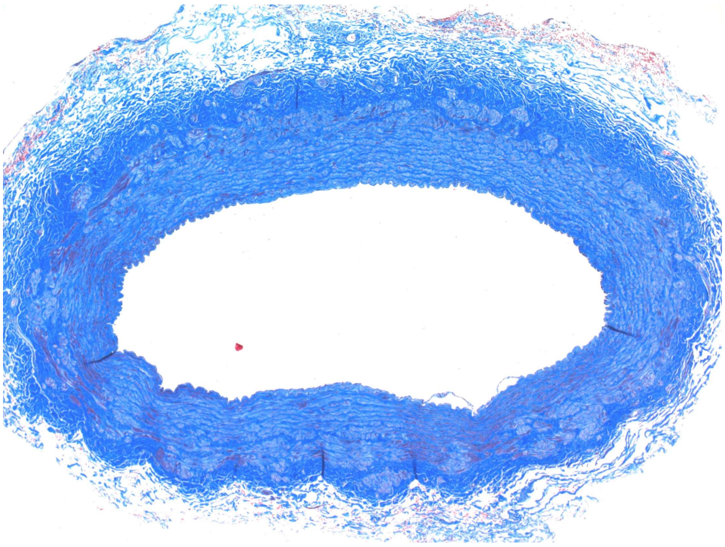

No.3 H&E (50X)

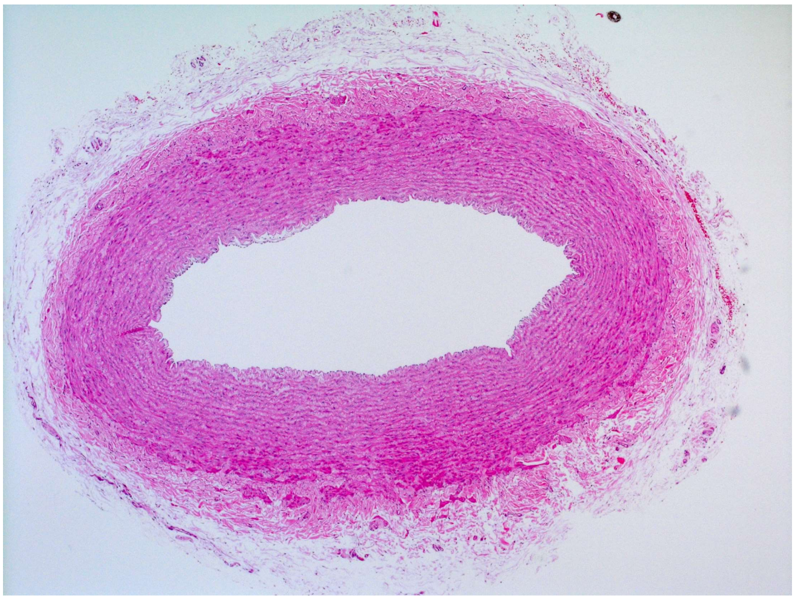

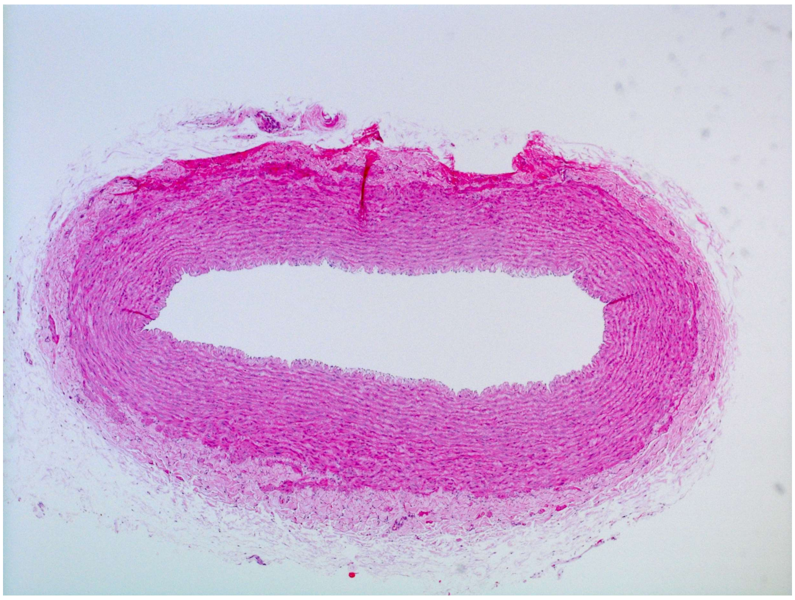

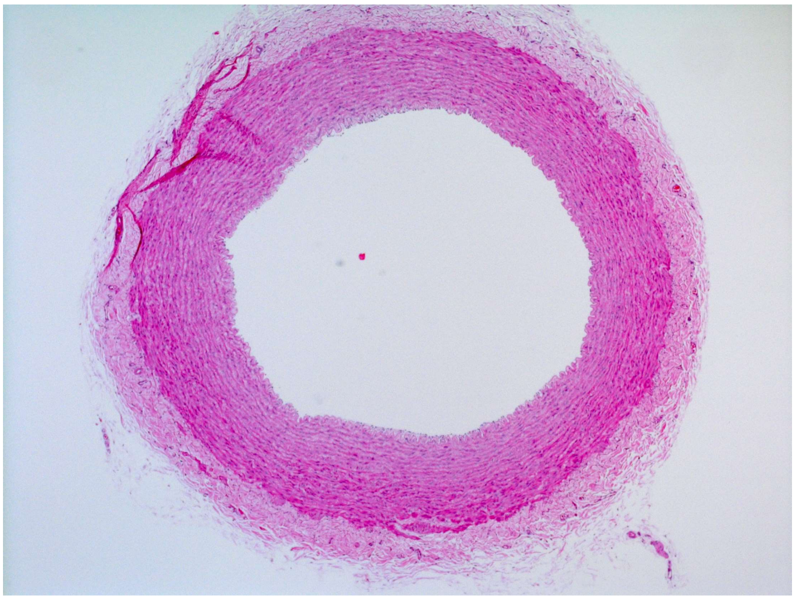

No.3 Masson's Trichrome (50X)

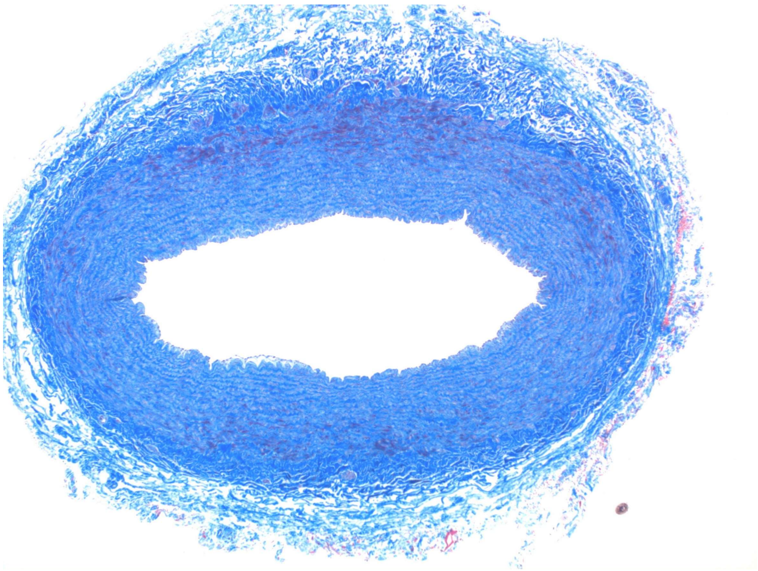

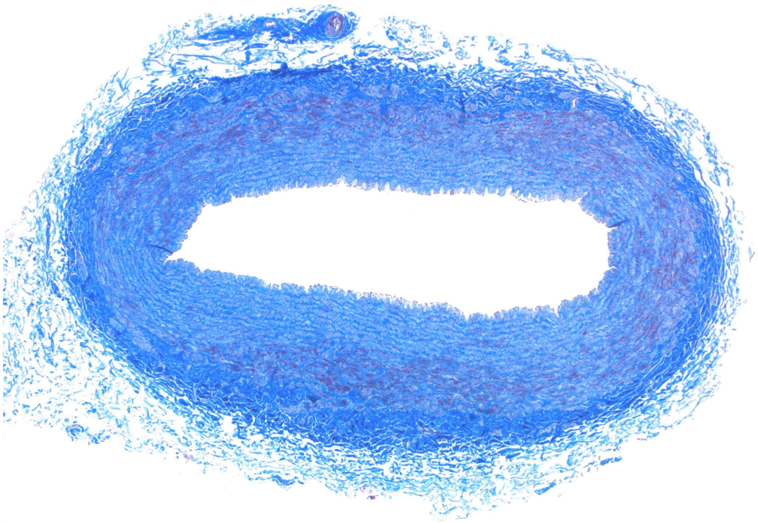

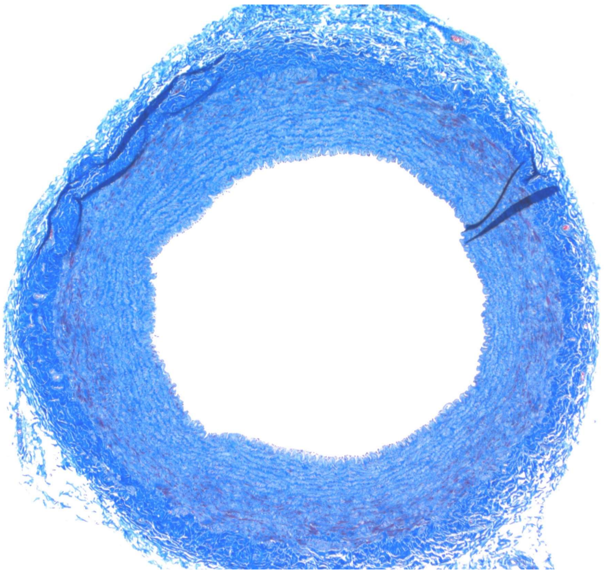

No.4 H&E (50X)

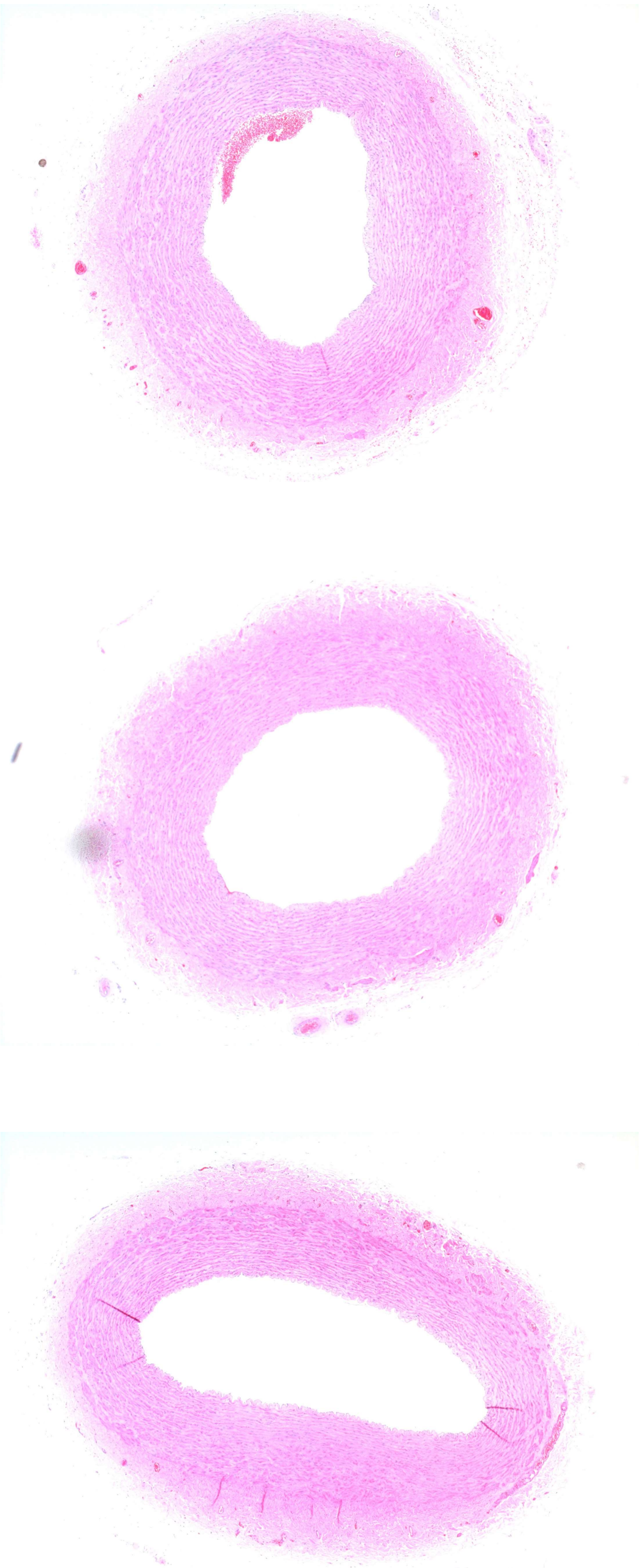

No.4 Masson's Trichrome (50X)

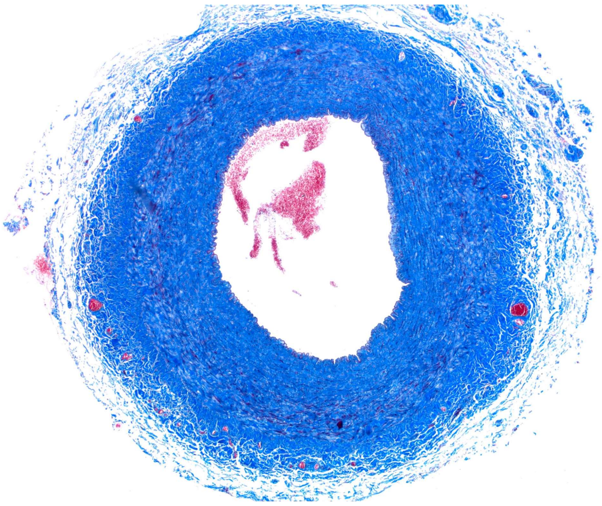

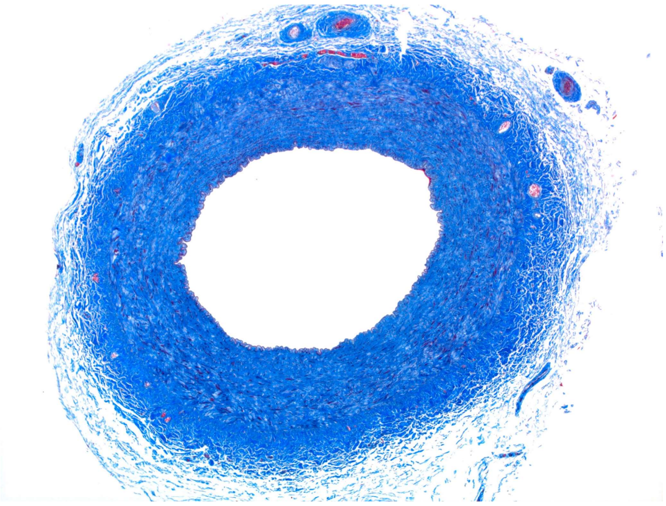

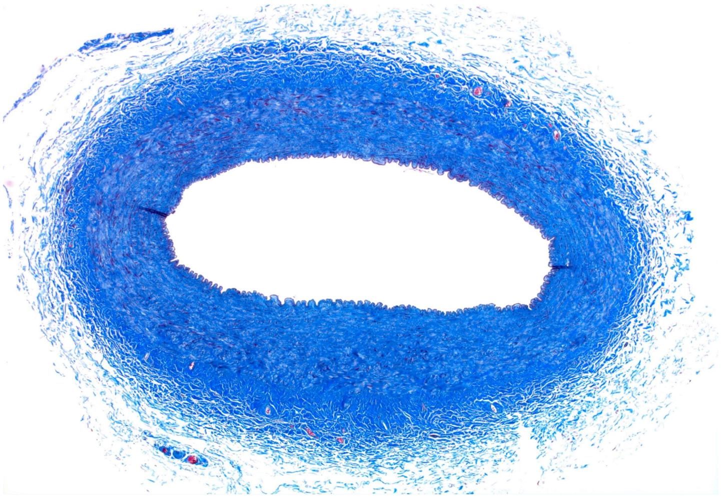

No.5 H&E (50X)

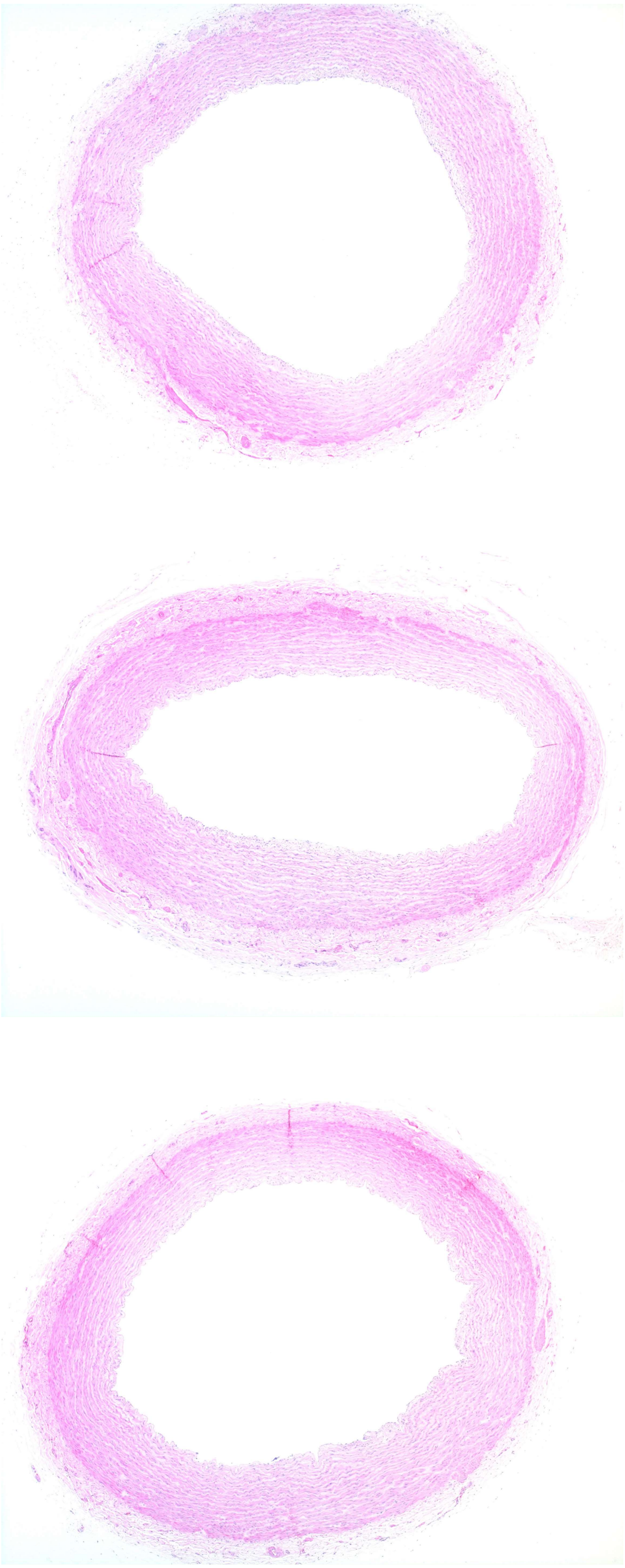

No.5 Masson's Trichrome (50X)

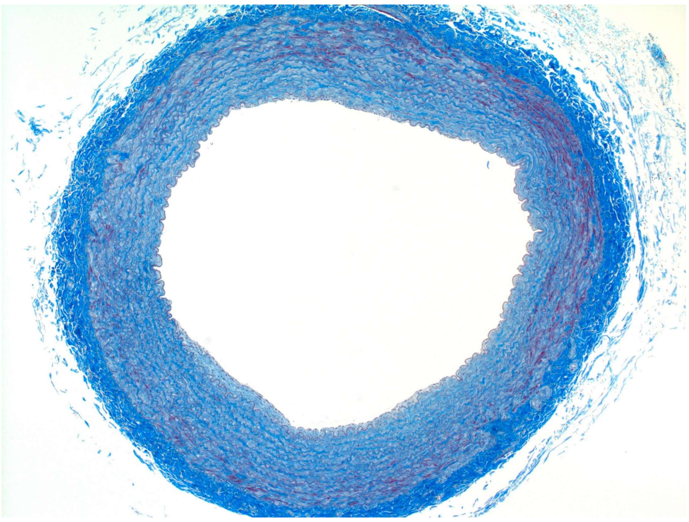

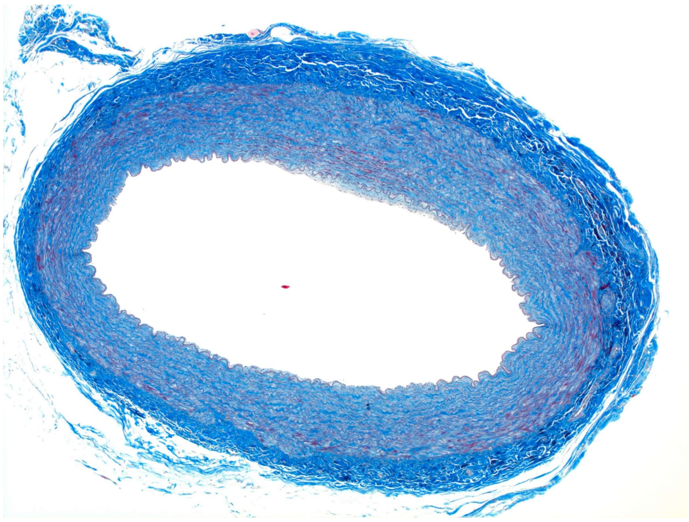

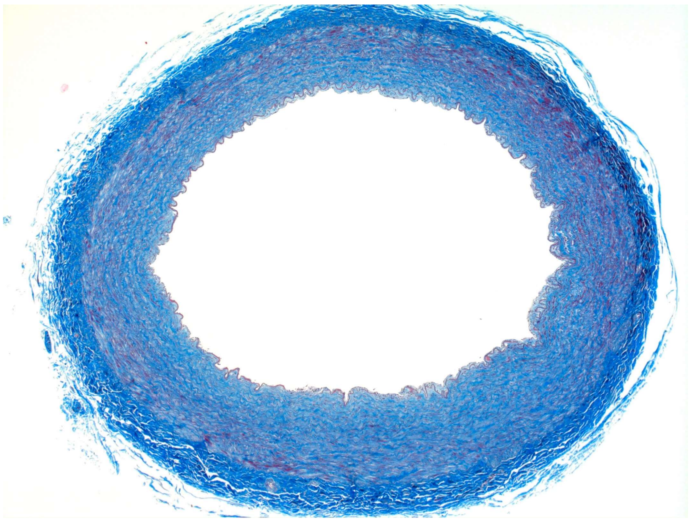

No.6 H&E (50X)

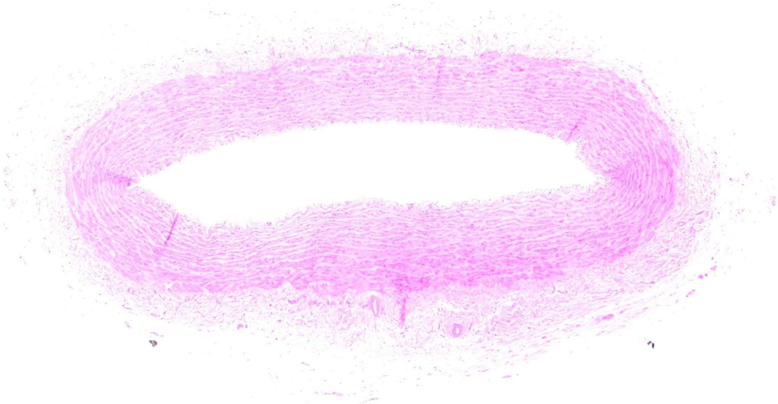

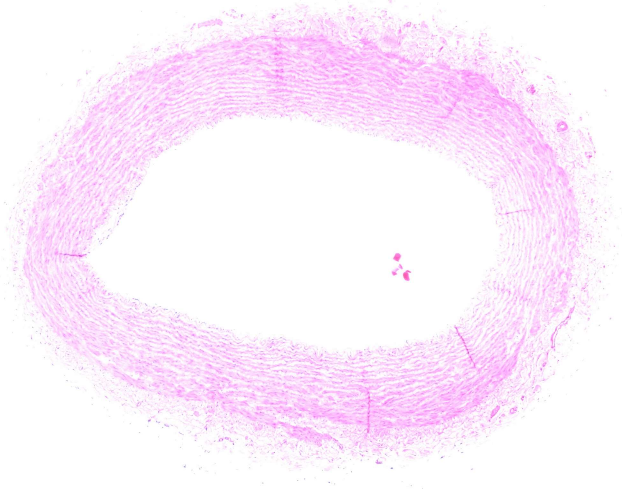

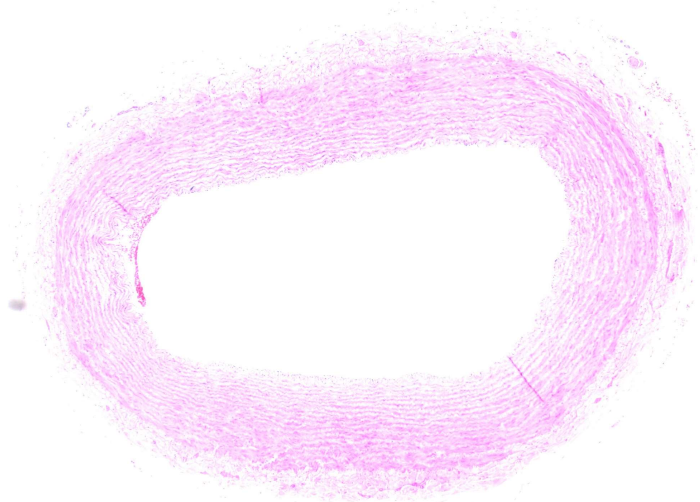

No.6 Masson's Trichrome (50X)

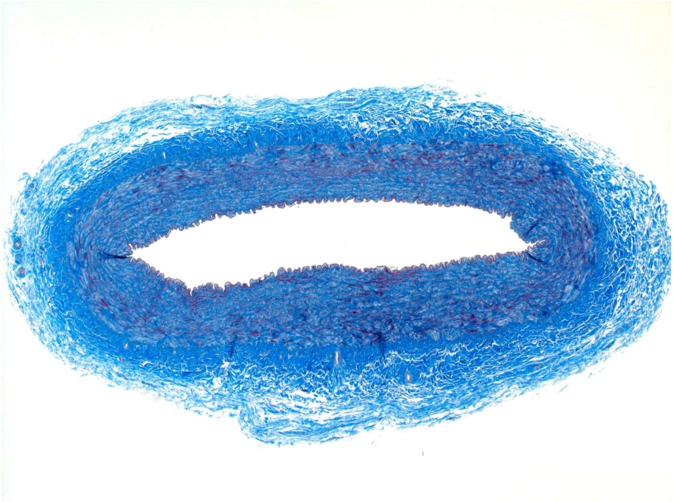

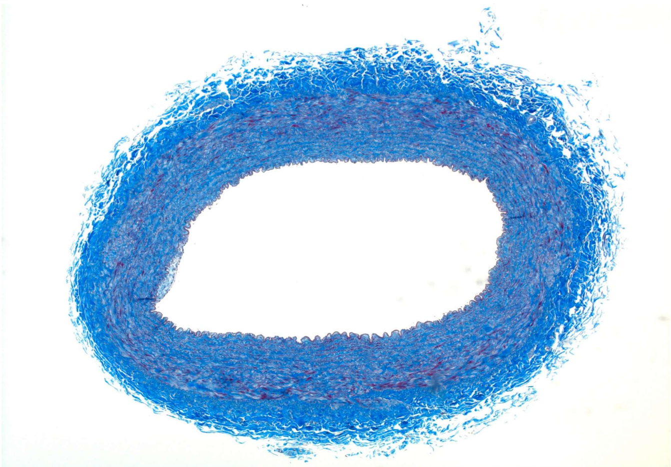

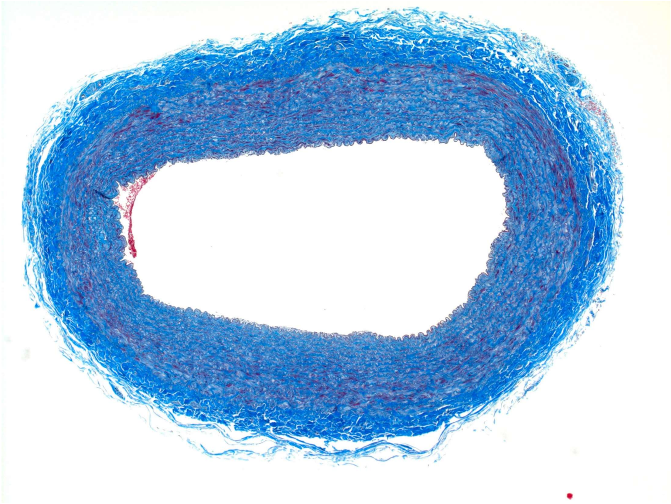

No.7 H&E (50X)

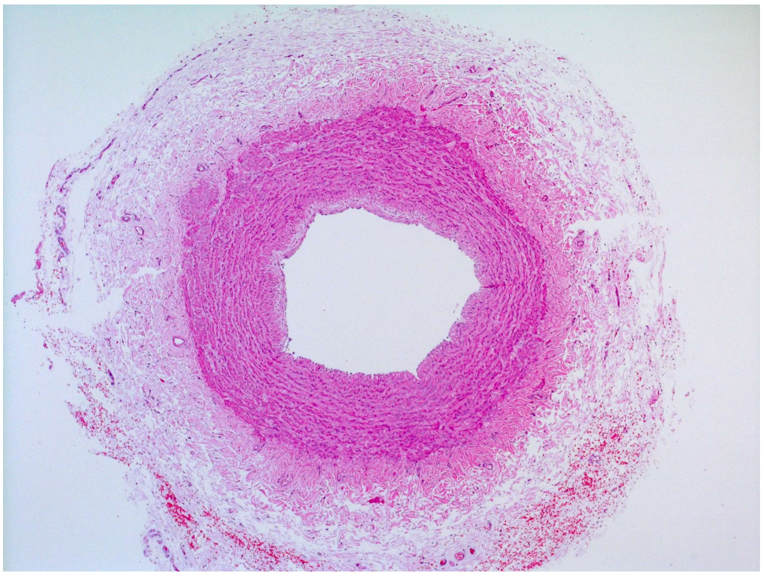

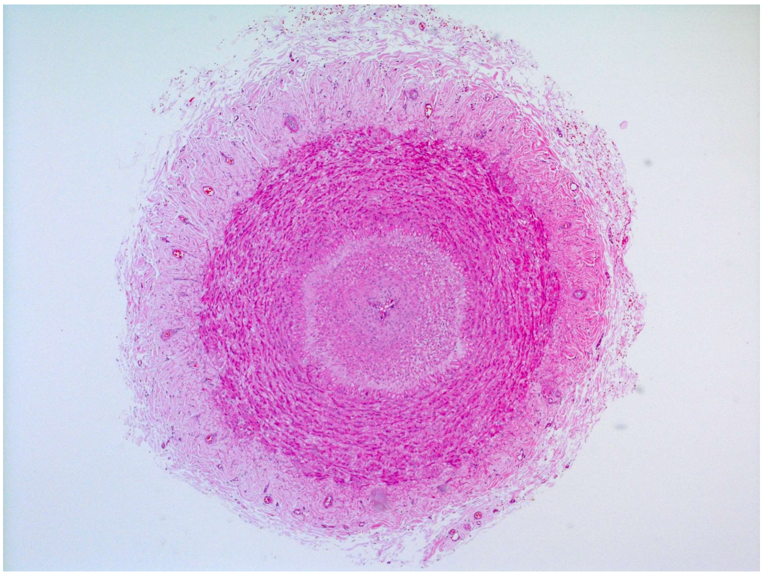

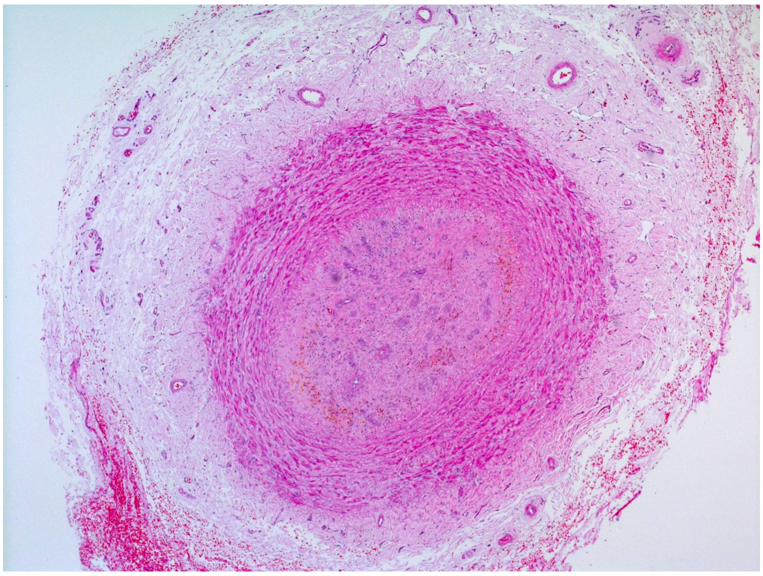

No.7 Masson's Trichrome (50X)

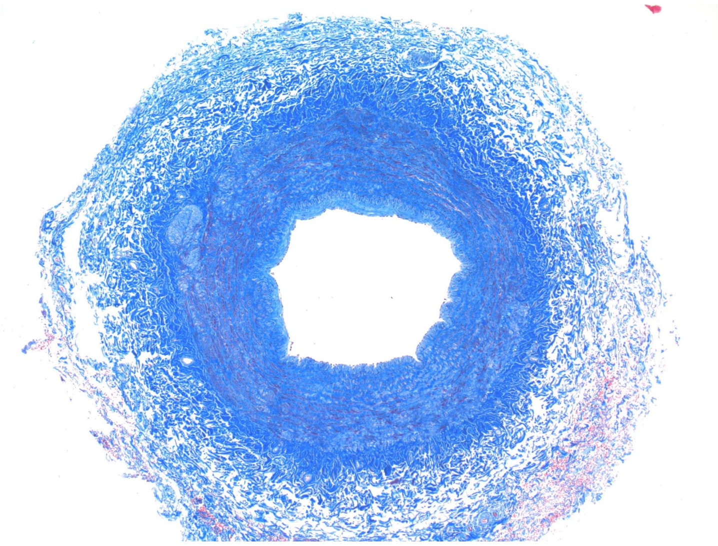

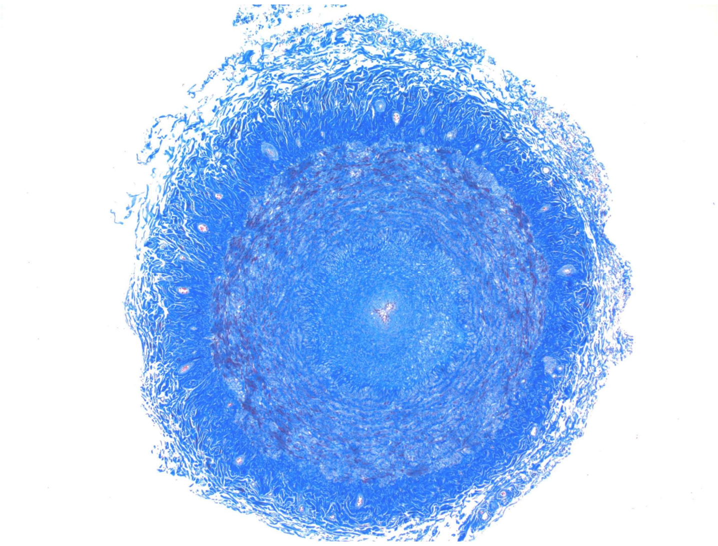

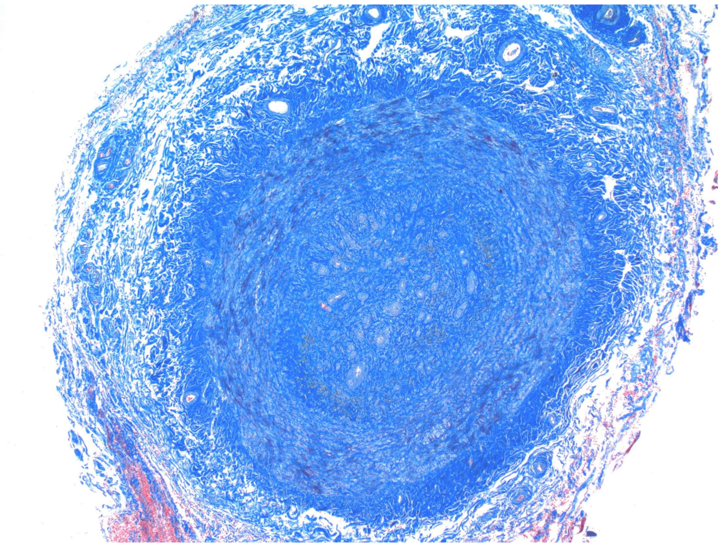

No.8 H&E (50X)

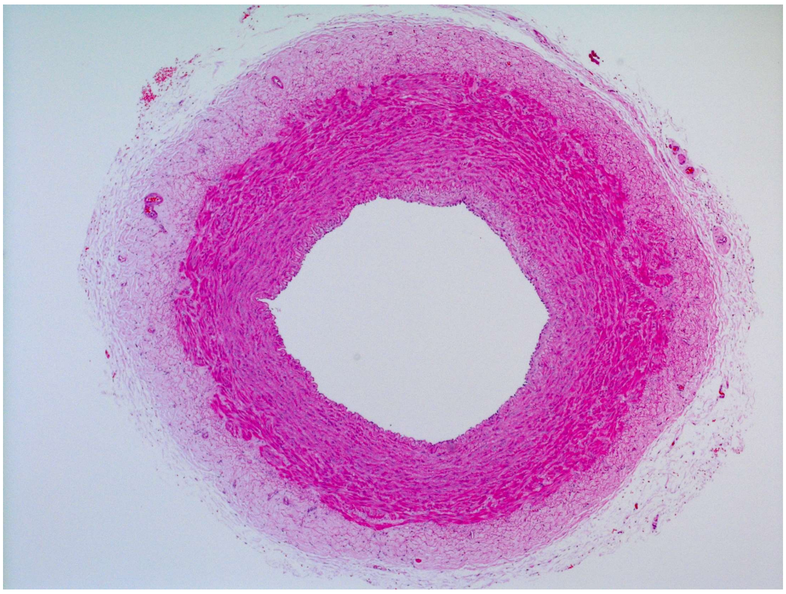

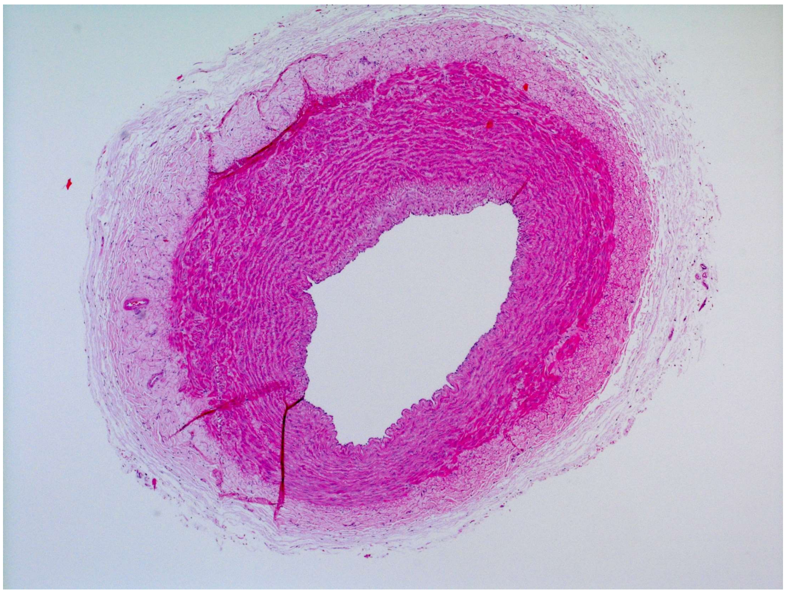

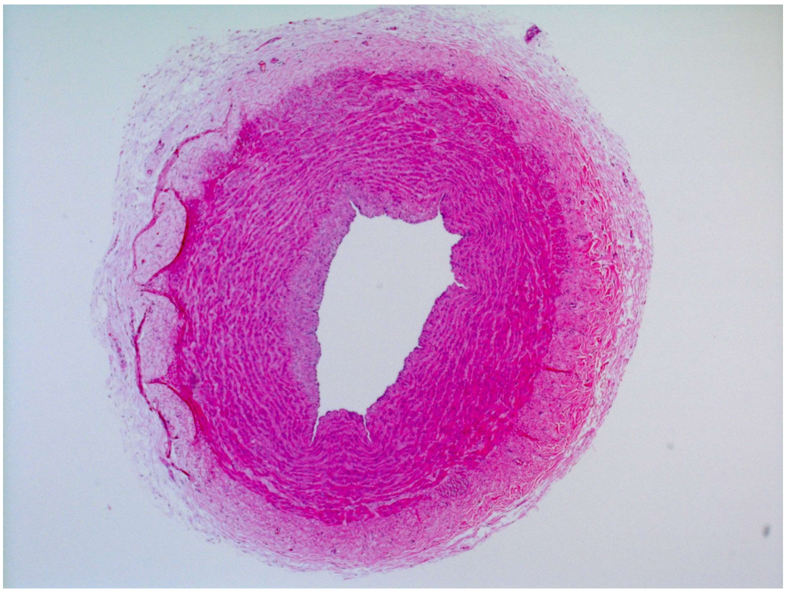

No.8 Masson's Trichrome (50X)

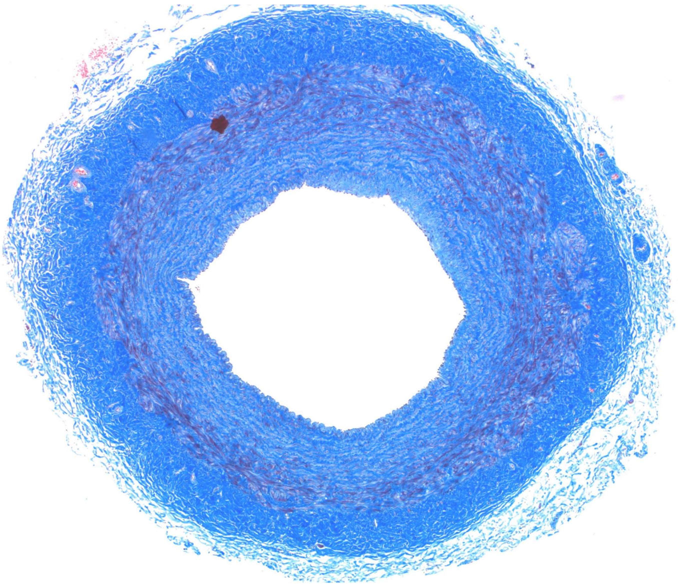

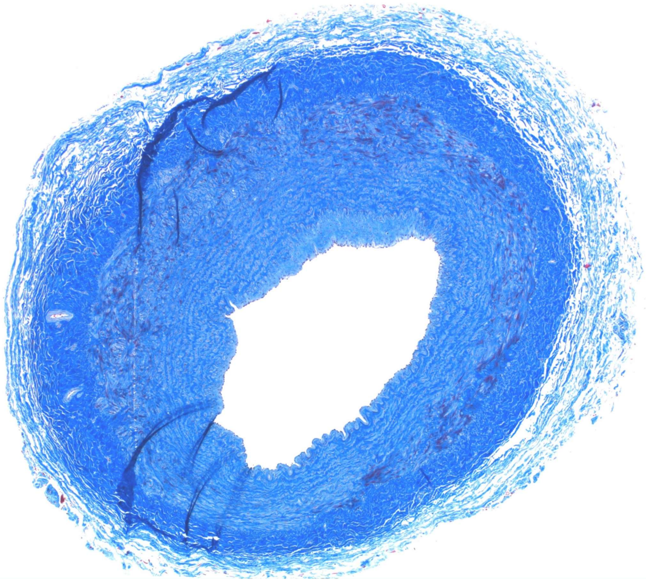

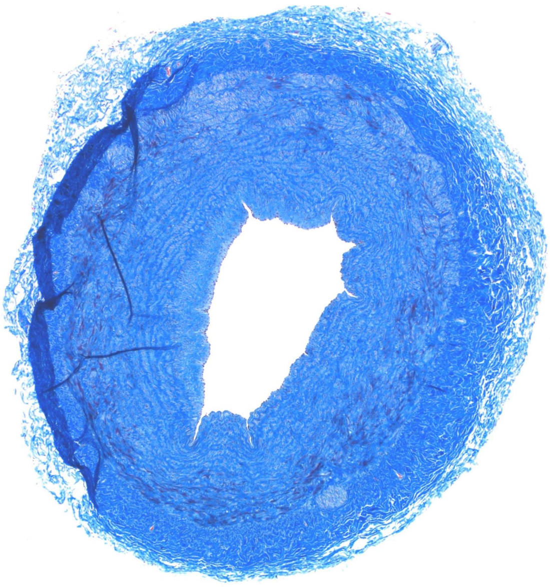

No.9 H&E (50X)

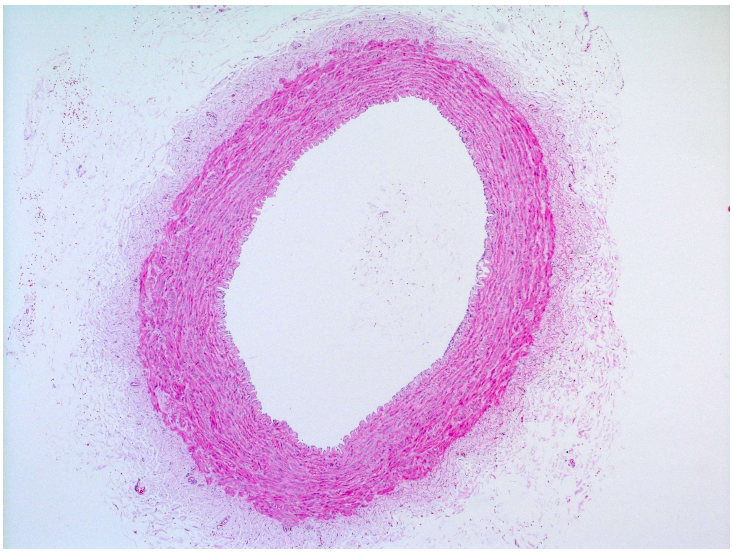

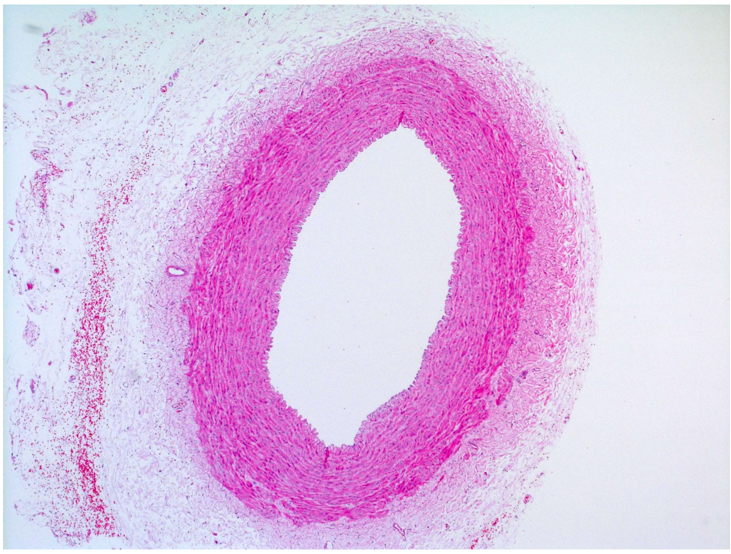

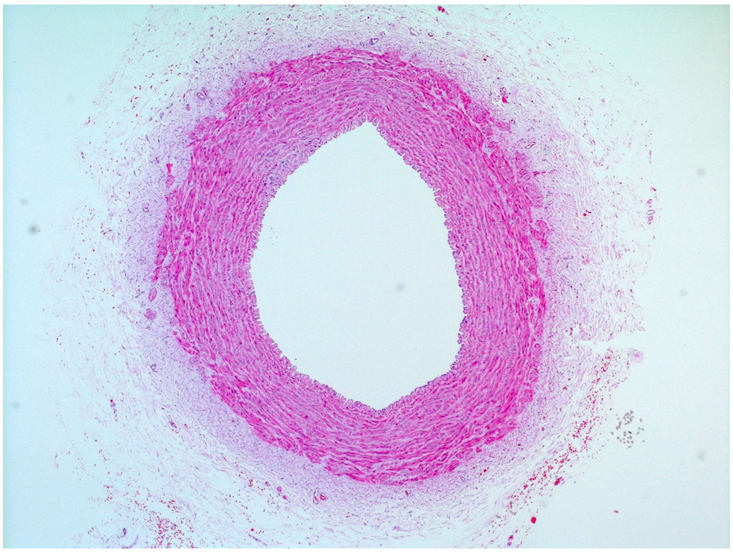

No.9 Masson's Trichrome (50X)

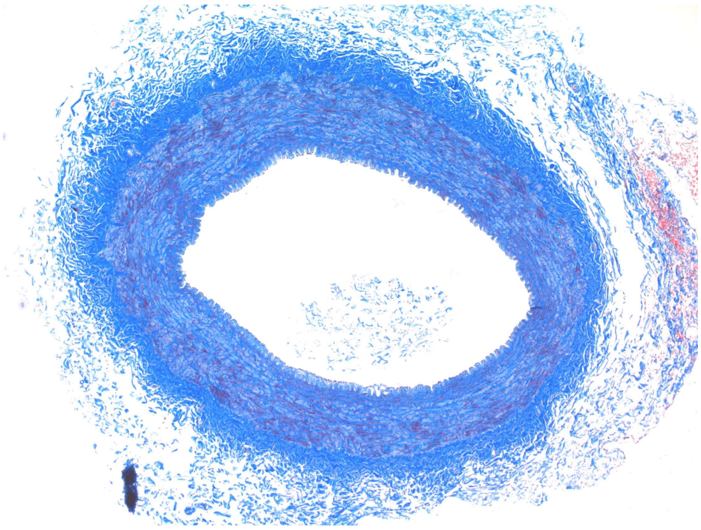

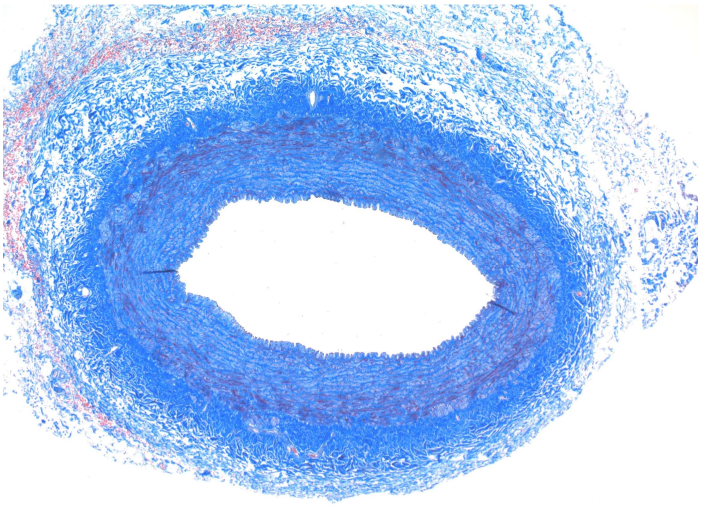

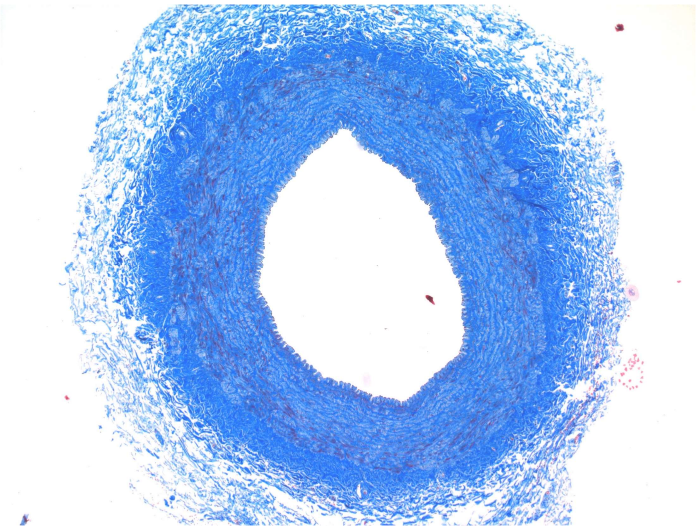

No.10 H&E (50X)

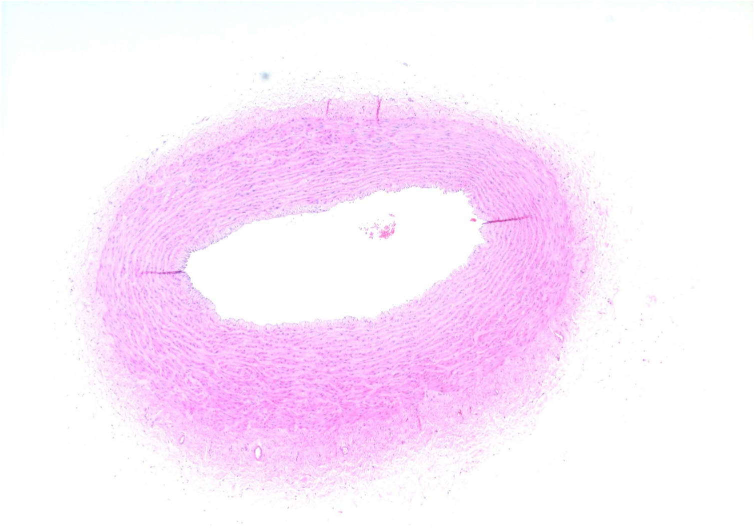

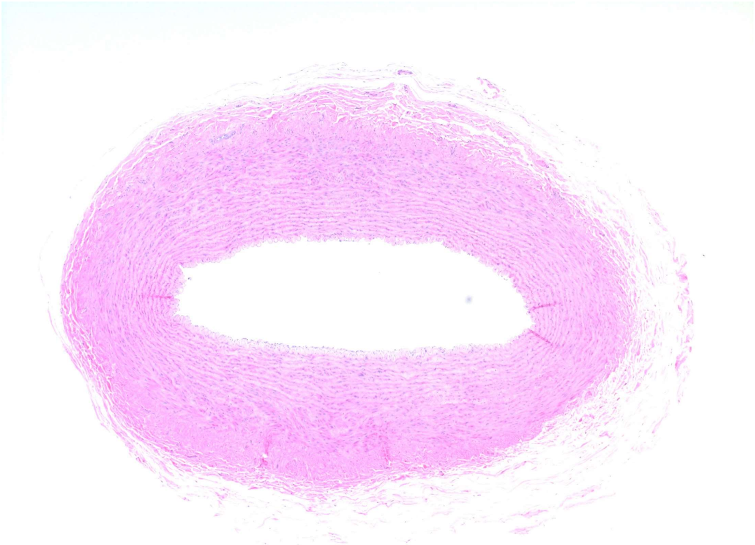

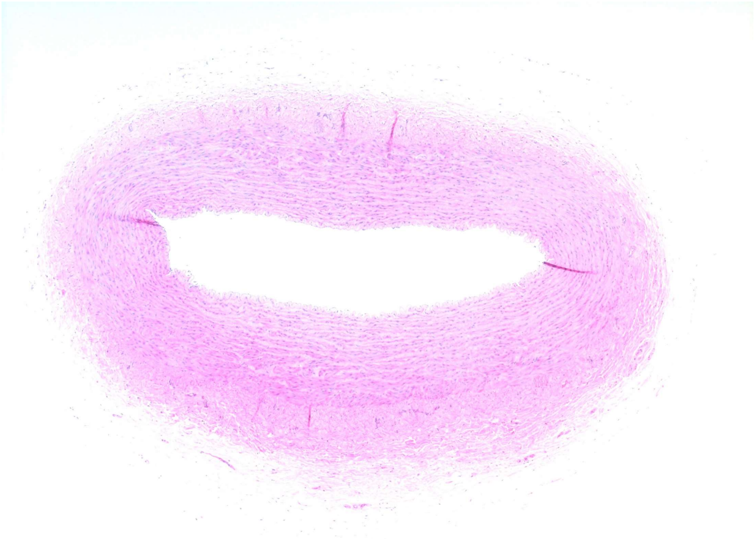

No.10 Masson's Trichrome (50X)

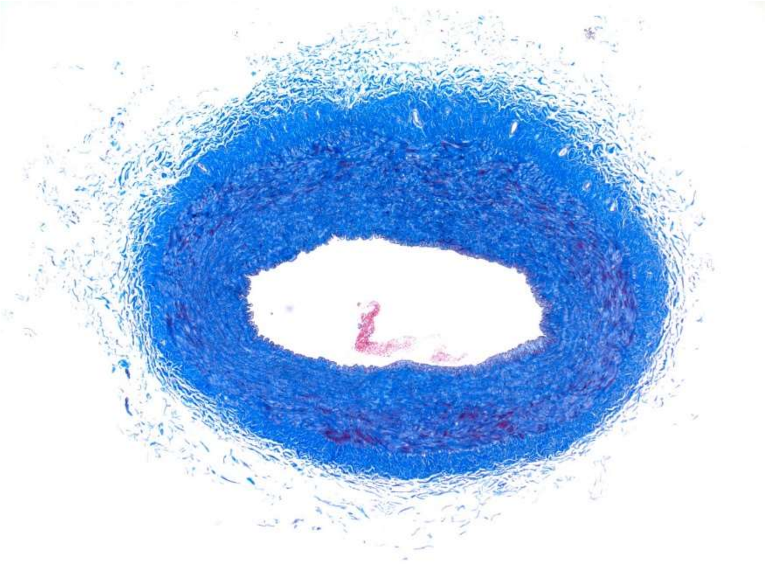

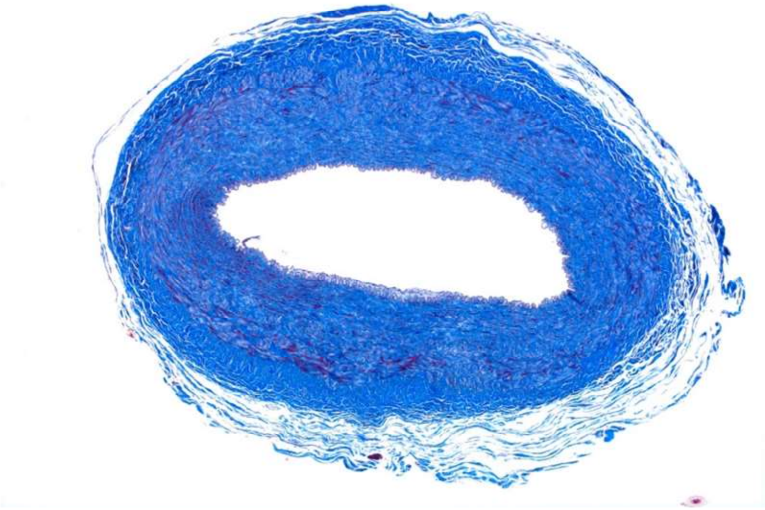

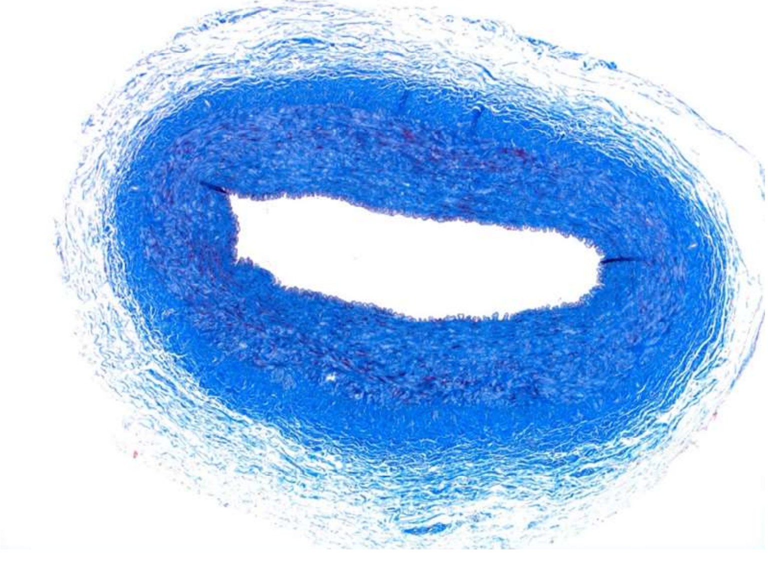

No.11 H&E (50X)

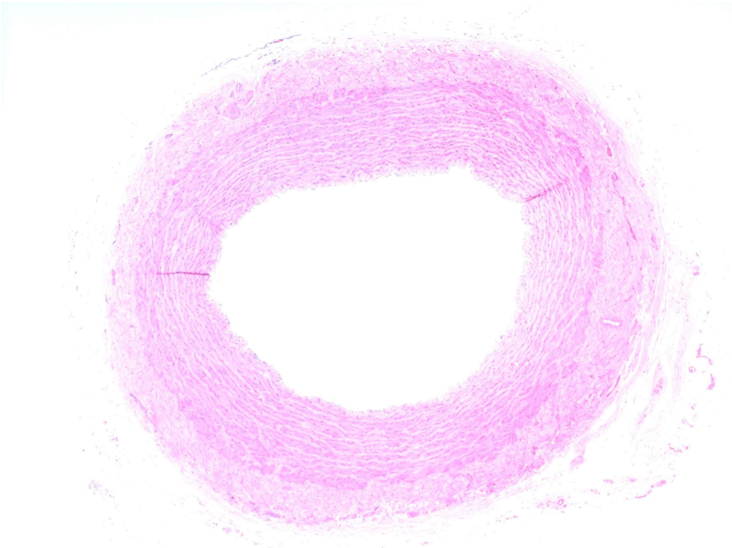

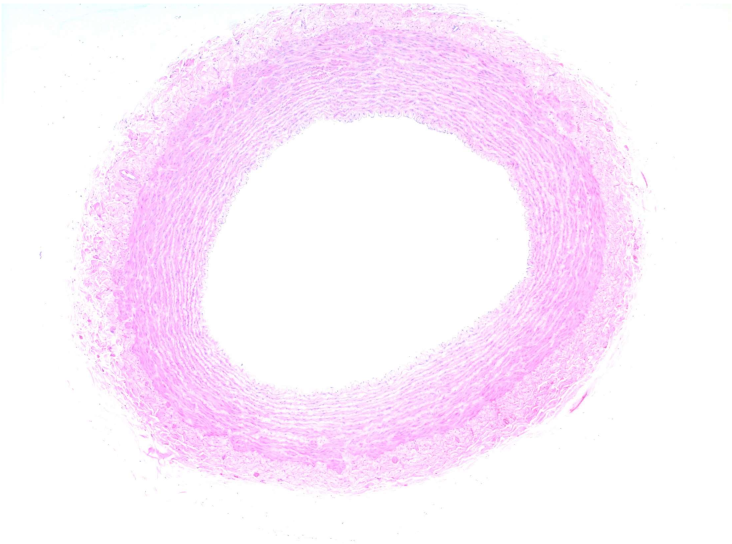

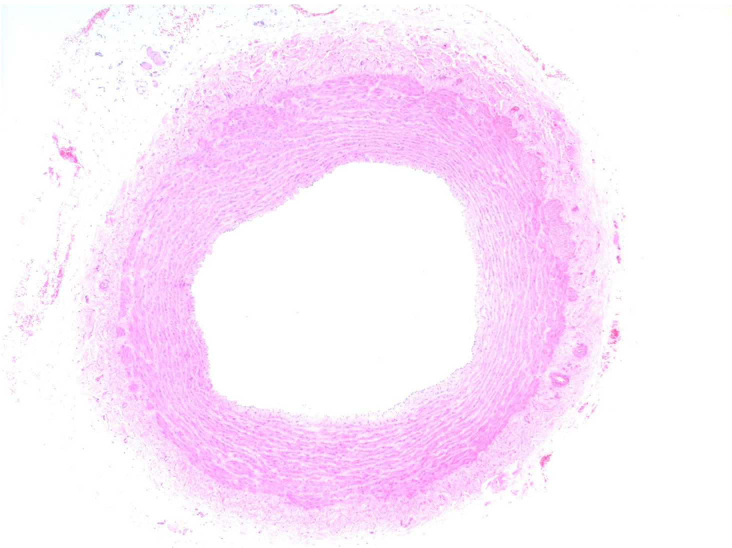

No.11 Masson's Trichrome (50X)

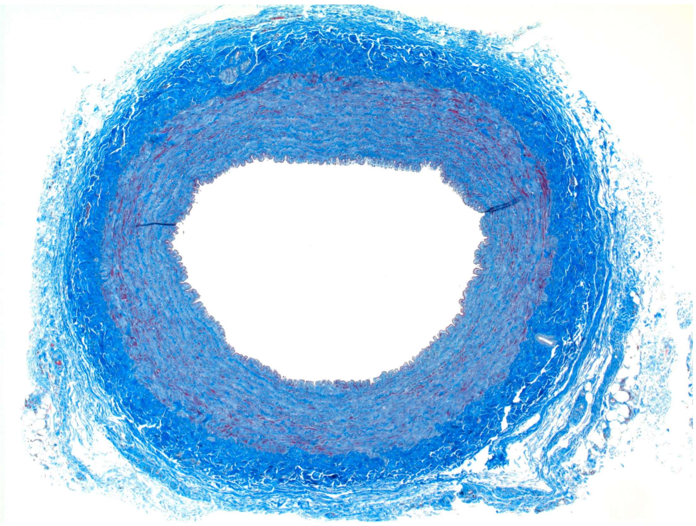

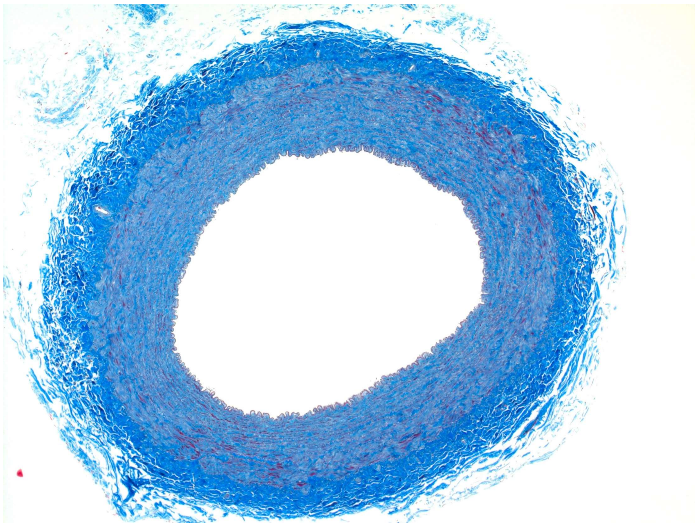

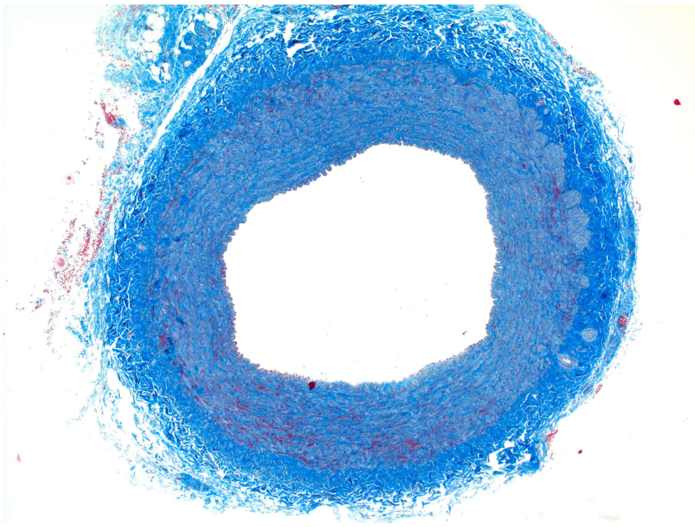

No.12 H&E (50X)

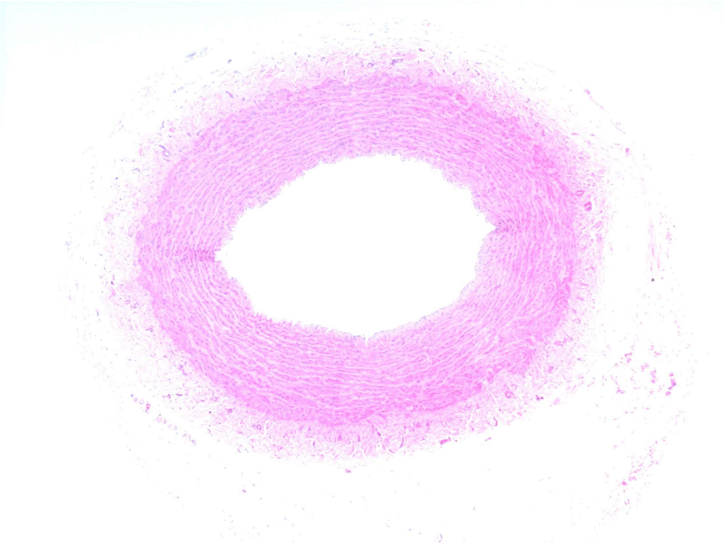

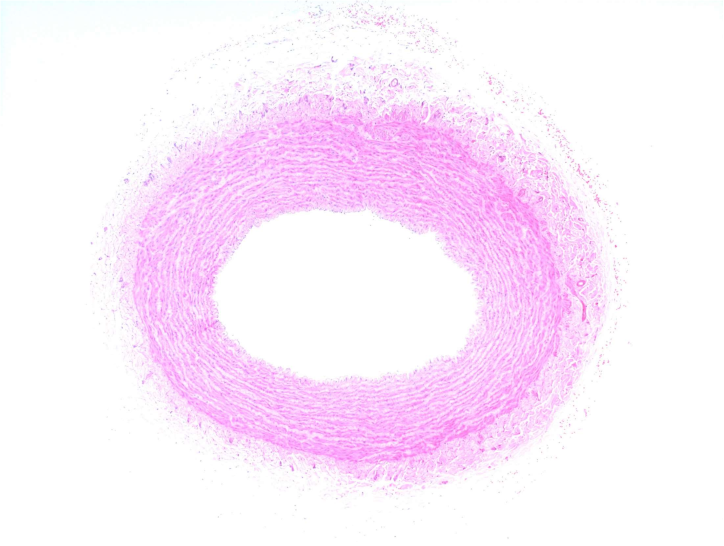

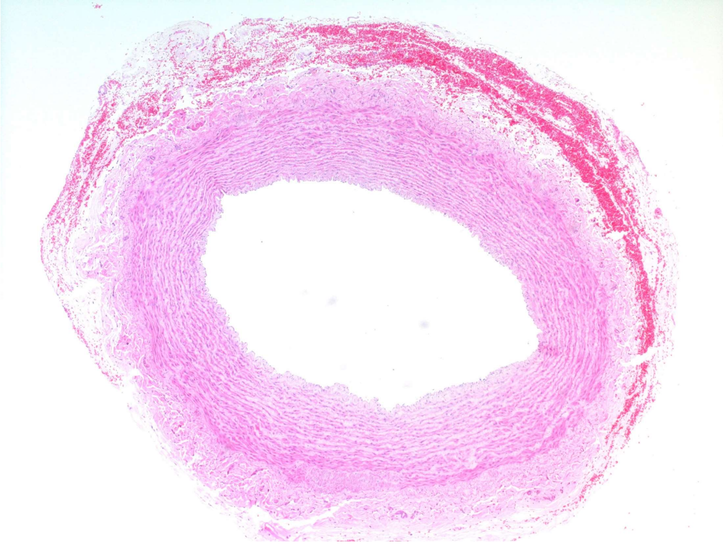

No.12 Masson's Trichrome (50X)

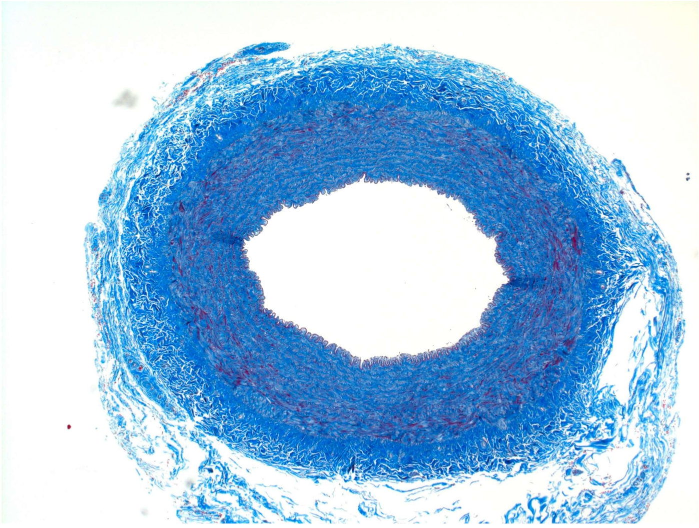

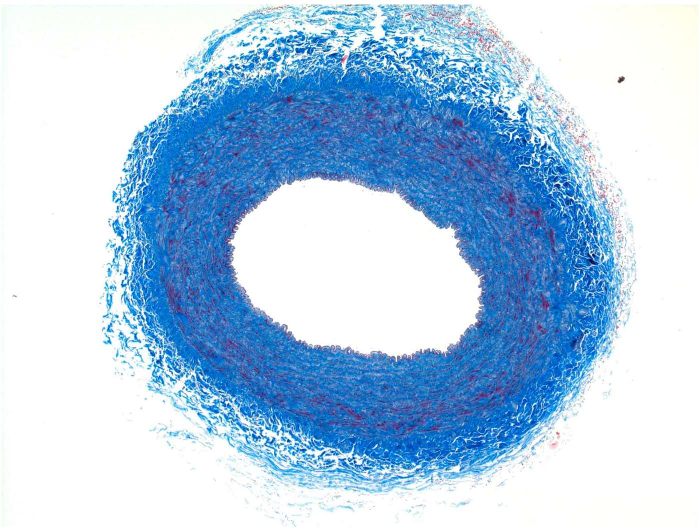

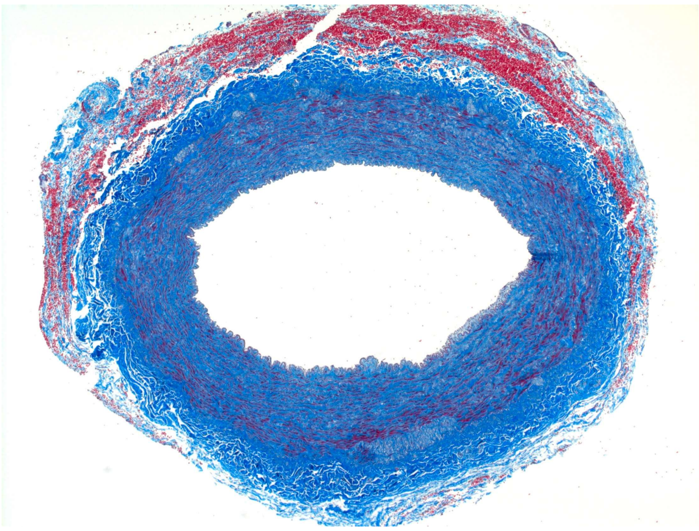

Supplement: S1 Fig — (PDF) [file pone.0276108.s001.pdf]
