# Supplementary material for: In vivo evaluation of histopathologic findings of vascular damage after mechanical thrombectomy with the Tromba device in a canine model of cerebral infarction
Source: PLoS One. 2022 Oct 14;17(10):e0276108. doi: 10.1371/journal.pone.0276108 (PMC9565453; doi:10.1371/journal.pone.0276108)
Supplement: S1 File — Evaluation of safety and efficacy of stent retrievers for treatment of ischemic stroke. (PDF) [file pone.0276108.s002.pdf]

English translation version

Final report

Evaluation of safety and efficacy of stent retrievers for treatment of ischemic stroke

Test number : 19-051-DE

Investigator : Sang-Woo Kim

Test period : 2019.01.01 ~ 2019.12.31

Test Requester : CG Bio Co., Ltd.

Commissioner: Seong-Hyun Kim

OSONG MEDIICAL IINNOVATIION FOUNDATIION LABORATORY ANIIMAL CENTER

Investigator Statement

Study Title : Evaluation of Safety and Efficacy of Stent Retriever for Treatment of Ischemic Stroke

This test was conducted according to the contents obtained by the Animal Experiment Steering Committee of the Osong Medical Innovation Foundation Laboratory Aninal Center (KBIO-IACUC-2019-051), and no other factors that could affect the test results were identified.

January 21, 2020

Experiment Manager: Sang-Woo Kim

Affiliation : Osong Medical Innovation Foundation Laboratory Aninal Center

Address: 123 Osongsaeng-ro, Osong-eup, Heungdeok-gu, Cheongju-si, Chungcheongbuk-do

Contact: ☎ 043-200-9851, 043-200-9805 (Fax)

E-mail: swkim@kbiohealth.kr

Exam Overview

**Trial Title**	Evaluation of Safety and Efficacy of Stent Retriever for Treatment of Ischemic Stroke
**Purpose of the test**	After constructing an animal model for blood vessel occlusion by making an autologous thrombus from a beagle dog, blood vessel patency was confirmed through stent retriever procedure. Clinical pathological analysis was also performed to confirm the indicators of hematology and blood biochemical examination and tissue Pathological analysis was performed to determine the degree of vascular endothelial damage and inflammation.

Test requester

**Name :**	CG Bio Co., Ltd
**Address :**	111, Suntec City 2, 52 Sagimakgol-ro, Seongnam-si, Gyeonggi-do
**Commissioner :**	Seong-Hyeon Kim
**Contact :**	☎ +82-31-748-8211<br>(Fax) +82-31-748-8222

Test Institution

**Name :**	Osong Medical Innovation Foundation Laboratory Aninal Center
**Address :**	123, Osongsaengsaeng-ro, Osong-eup, Heungdeok-gu, Cheongju-si, Chungcheongbuk-do
**Investigator :**	Sang-Woo Kim
**Contact :**	☎ +82-43-200-9851<br>(Fax) +82-43-200-9805

**Test Schedule**	**May 12, 2019: Approval of animal test protocol**
	**June 12, 2019: Bringing in laboratory animals**
	**June 19, 2019 : End of acclimatization of laboratory animals**
	**June 20, 2019 Autologous thrombus production and stent retriever operation 1**
	**June 28, 2019 : Autologous thrombus production and stent retriever operation 2~3**
	**July 11, 2019: Autologous thrombus production and stent retriever operation 4**
	**July 19, 2019: Autologous thrombus production and stent retriever operation 5~6**
	**July 22, 2019: Reconfirmation of vascular patency one month after surgery, autopsy 1**
	**July 29, 2019: Reconfirmation of vascular patency 1 month after surgery, autopsy 2~3**
	**August 09, 2019 : Reconfirmation of vascular patency 1 month after surgery, autopsy 4**
	**August 16, 2019: Reconfirmation of vascular patency 1 month after surgery, autopsy 5~6**
	**August 23, 2019: Autologous thrombus production and stent retriever operation 7~8**
	**September 20, 2019 : Autologous thrombus production and stent retriever operation 9~10**
	**September 23, 2019: Reconfirmation of vascular patency 1 month after surgery, autopsy 7~ 8**
	**September 26, 2019 : Autologous thrombus production and stent retriever operation 11~ 12**
	**October 23, 2019: Reconfirmation of vascular patency 1 month after surgery, autopsy 9~ 12**
	**November 04, 2019: Histopathological analysis conducted**
	**December 02, 2019: Conducted histopathological examination**
	**January 17, 2020 : Submission of draft final report**
	**January 21, 2020 : Submission of final report**

Major test personnel

Field	Name	Responsibilities
**Breeding management**	Jung-Hyun Kim, DVM, Ph.D	Animal breeding manager
**Animal Testing**	Sang-Woo Kim DVM, MS	Animal testing director
	Rae-Hyung Yu DVM, Ph.D	Animal test manager
	Ji-Young Yoon, MS	Animal test manager
	Hyung-Ho Moon MS	Animal test manager
	Jong-Min Kim MS	Animal test manager
	Sung-Hoon Kim MS	Animal test manager
	Seon-Won Lee MS	Animal test manager
**autologous blood clot production**	Jong-Min Kim MS	Animal test manager
	Sung-Hoon Kim MS	Animal test manager
**Stent Retriever Procedure**	Sang-Hun Lee MD, Ph.D [a)]	Responsible for the procedure
	Sang-Woo Kim DVM, MS	Animal testing director
**Person in charge of checking blood vessel patency**	Sang-Hun Lee MD, Ph.D [a)]	Responsible for the procedure
	Jong-Min Kim MS	Animal testing manager
**clinical pathology analysis**	Jin-Ah Kim MS	Clinical pathologist
**Histopathological analysis**	Ji-Hyun Choi MS	Responsible for histopathology
**histopathology reading**	Woo-Chan Son DVM, Ph.D [b)]	Histopathology reading

a) Korea University Ansan Hospital, 123, Jeokgeum-ro, Danwon-gu, Ansan-si, Gyeonggi-do

b) Laboratory Animal Pathology Laboratory, Asan Medical Center, 88, Olympic-ro 43-gil, Songpa-gu, Seoul

1. Summary

In this test, an autologous thrombus was made using 12 beagle dogs and blood vessel occlusion was induced, and then a control device (name: Solitaire ™ FR) or a test device (stent retriever : Tromba) was used according to the group composition. Angiography was used to confirm the patency of the occluded blood vessels, and clinical pathological analysis was performed to compare and evaluate indicators in hematology and blood biochemistry. In addition, one month after the procedure, the patency of the blood vessels was reconfirmed using angiography, and the right carotid artery through which the stent retriever passed was excised and histopathological analysis was performed.

1) As a result of angiography, it was confirmed that vascular occlusion was stably induced as blurring of blood flow was blocked in the blood vessels injected with autologous thrombi.

2) It was confirmed that both the control device (Solitaire™ FR) and the test device (stent retriever: Tromba) were deployed without any problem in the occluded blood vessel.

3) As a result of vascular patency evaluation, complete patency (vessels in which diameter reduction was not observed due to autologous thrombosis) between the control group and the experimental group was confirmed to be at a similar level, and both the control group and the control group maintained the patency of the blood vessels 1 month after the procedure. .

4) As a result of clinical pathology, no significant difference was observed in all items of hematology and blood biochemistry between the control group and the experimental group.

5) As a result of histopathological examination, a weak (+2) degree of intimal proliferation was observed in the control group and the experimental group. Also, in the experimental group, loss of endothelial cells and damage to the mesenteric membrane were observed, but only slightly (+1).

Combining the above results, it was possible to observe that all blocked blood vessels

were opened due to the stent retriever procedure with the control device (Solitaire™ FR) and the inspection device (central circulatory system embolization catheter), and it was confirmed that there was no damage.

2. Materials and Methods

1) Test equipment

(1) Name: Tromba thrombectomy device

(2) Usage: Catheter, Thrombus Retriever

(3) Supplier: CG Bio Co., Ltd.

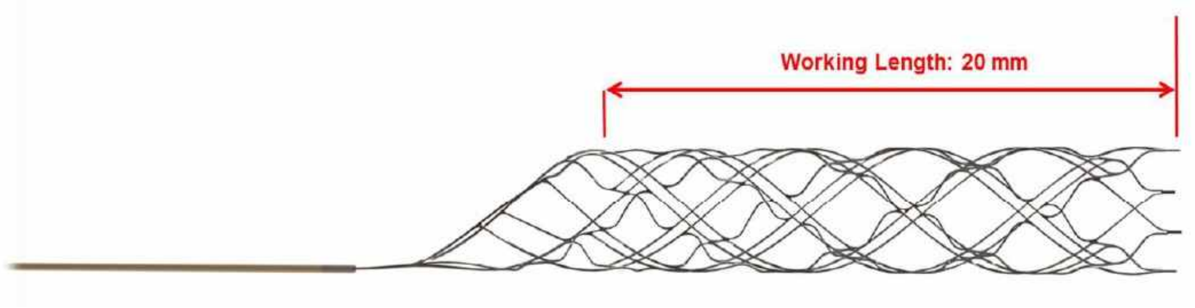

2) Compatible devices

(1) Name: Solitaire™ FR

(2) Usage: Percutaneous Catheter

(3) Source: CG Bio Co., Ltd.

3) Test system

(1) Species and strains: Beagle dog

(2) Gender: Male

(3) 5 months of age at the time of acquisition

(4) Weight: 6.0~7.7 kg

(5) Number of animals: 12

(6) Source: Marshall BioResources / Friendship BSC

(7) Reason for choosing the test system

In order to evaluate the effectiveness of the control device and the test device stent retriever after inducing blood vessel occlusion, the arterial vessel size of the beagle dog was judged to be the most appropriate. [Manik Mehra, J NeuroIntervent Surg, 2012 ;4:307-313)

(8) Quarantine and Purification

When acquiring animals, inspection and quarantine were conducted referring to the report provided by the supplier. During the 7-day acclimatization period, general symptoms were observed once a day, and only healthy animals were provided for the experiment.

4) breeding environment

(1) Environmental conditions

In this experiment, temperature 23±3℃, relative humidity 55±15%, ventilation frequency of 10~20 times/hr, lighting time 12 hours (lights on at 8am ~ lights out at 8pm), illuminance of 150~300 Lux was used in the medical industry. It was held in the dog breeding room of the Institute for Laboratory Animals Center. All the experimenters wore work clothes and protective equipment that were autoclaved (121°C, 20 minutes).

(2) Identification of cage, breeding density and breeding box

One animal/cage was bred in a cage (0.9 m^2) wider than the AAALAC guidelines (0.74 m^2), and a personal identification card with the test number and animal number was attached to the breeding cage. Kong classic dog toy was provided for environment enrichment

(3) Feed and water feeding method

For the feed supplied to the test, a solid feed for test animals (2925 Telan Global 25% Protein Dog Diet). Raw materials produced at the RO water (reverse osmosis distilled water) manufacturing and supply facility were sterilized using sodium hypochlorite (NaOCl) and UV equipment, and then freely consumed through an automatic water supply nozzle.

(4) Compliance with Animal Experimental Ethics Regulations

This test was conducted at the Osong Advanced Medical Industry Promotion Agency's Laboratory Animal Center and other public institutions under the Ministry of Health and Welfare that received AAALAC accreditation. The contents of the application for approval of animal testing approved by IACUC (KBIO-IACUC-2019-051) were followed.

5) Test group composition

Group	Device	Number	No	Target artery	F/U period
Control group	Solitaire FR	6	#1	Rt-Occipital	1 Month
			#4	Rt-Occipital	
			#6	Rt-Superficial	
			#8	Rt-Occipital	
			#10	Rt-Occipital	
			#12	Rt-Superficial	
Experimental group	Tromba	6	#2	Rt-Linguofacial	1 Month
			#3	Rt-Linguofacial	
			#5	Rt-Occipital	
			#7	Rt-Occipital	
			#9	Rt-Occipital	
			#11	Rt-Superficial	

6) Experimental method

(1) Autogenous thrombus formation

- To recreate an environment similar to acute human stroke, red thrombi were designed in the occluded arteries.

- Thrombi were created by mixing 10 mL of autologous blood of the animal with 25 IU of bovine thrombin solution (Dade Behring, Newark, NJ, USA).

-After mixing, the blood was injected into a silicone tube and incubated for 120 minutes at room temperature.

-At the end of the incubation, thrombi were stored for 24 hours under refrigeration at 4°C.

-Each prepared thrombus, which was 10 mm in length, was inserted into a silicone tube with saline and prepared for injection.

(2) laboratory animal anesthesia

- The procedures were conducted with the canines under general anesthesia.

- General anesthesia was induced by intramuscular injection of 5 mg/kg tiletamine/zolazepam (ZoletilTM, Virbac Korea, Seoul, South Korea) and 2 mg/kg Xylazine (Rompun, Bayer Korea, Ansan, South Korea) and maintained with isoflurane (<3%).

- Vital parameters, such as arterial blood pressure, heart rate, and end tidal carbon dioxide levels, were continuously recorded.

- All procedures were conducted according to international guidelines and were approved by the responsible local authorities.

(3) Construction of Vascular Occlusion Model

- A short 6F catheter sheath (Terumo, Tokyo, Japan) was inserted into the right common femoral artery and continuously flushed with physiological saline.

- Selective intra-arterial digital subtraction angiography (DSA) was performed on a monoplane high-resolution angiography system (Artis Zee Multi-Purpose, Siemens, Erlangen, Germany).

- After initial angiography of the external carotid artery, one vessel with good access was selected for occlusion. For selective thromboembolization, the preformed thrombus was injected into a 6F intermediate catheter (Envoy DA, DePuy Synthes Companies, Warsaw, IN, USA) positioned in the target vessel.

- After thrombus application, the intermediate catheter was removed for 15 minutes to restore arterial flow and allow embedding of the thrombus.

(4) Stent retriever procedure

- The microcatheter was passed through the occlusion-guided blood vessel in the angiography room in the animal laboratory.

- A control device or test device stent retriever was inserted into the microcatheter.

- In the unfolded state, the recovery time was maintained for 5 minutes.

- The microcatheter and stent retriever were withdrawn together.

(5) Vascular patency evaluation

- Diameter reduction due to autologous thrombus and distal vessel reperfusion after stent retriever surgery in occluded vessels was evaluated.

- According to the degree of patency of the occluded blood vessels, they were classified into complete patency, almost complete patency, partial patency, and no patency.

- One month after the stent retriever procedure, angiography was performed again to reconfirm the patency of the blood vessels.

(6) Clinicopathological Analysis Hematology Tests

CBC	WBC (White blood cell count)	$10^3/\mu L$	Flowcytometry
	RBC (Red blood cell count)	$10^6/\mu L$	Flowcytometry, Isovolumetry
	HCT (Hematocrit)	%	(RBC×MCV)÷10
	HGB (Hemoglobin conc.)	g/dL	Modified CN met-Hb method
	MCV (Mean corpuscular volume)	fL	Histogram
	MCH (Mean corpuscular Hb)	Pg	(HGB÷RBC)×10
	MCHC (Mean corpusc. Hb conc.)	g/dL	[HGB÷(RBC×MCV)]×1,000
	PLT (Platelet)	$10^3/\mu L$	Flowcytometry
WBC Differential Count	%NEU (Neutrophil)	%	(NEUT x 100)÷WBC
	%LYM (Lymphocyte)	%	(LYM x 100)÷WBC
	%MONO (Monocyte)	%	(MONO x 100)÷WBC
	%EOS (Eosinophil)	%	(EOS x 100)÷WBC
	%BASA (Basophil)	%	(BASO x 100)÷WBC
	%LUC (Large unstained cells)	%	(LUC x 100)÷WBC
	#NEU (absolute NEU)	$10^3/\mu L$	Flowcytometry, Peroxidase stain
	#LYM (absolute LYM)	$10^3/\mu L$	Flowcytometry, Peroxidase stain
	#MONO (absolute MONO)	$10^3/\mu L$	Flowcytometry, Peroxidase stain
	#EOS (absolute EOS)	$10^3/\mu L$	Flowcytometry, Peroxidase stain
	#BASO (absolute BASO)	$10^3/\mu L$	Flowcytometry, Peroxidase stain
	#LUC (absolute LUC)	$10^3/\mu L$	Noise-Lymph Histogram
RET	RET (Reticulocyte)	%	RBC x (RET÷100) x 1000
	RET absolute	$10^9/\mu L$	Flowcytometry, Iso-volumetry, Absorbance(RNA content)
	ESR (Erythrocyte sedimentation rate)	mm	Westergren method

(7) Clinicopathological analysis Blood biochemical test

AST (Aspartate aminotransferase)	U/L	IFCC
ALT (Alanine aminotransferase)	U/L	IFCC
ALP (Alkaline phosphatase)	U/L	SCE (DEA buffer)
GGT (Gamma-glutanyltransferase)	U/L	IFCC
LDH (Lactate dehydrogenase)	U/L	SCE
BIL (Total bilirubin)	mg/dL	Diazotized sulf. Acid
PRO (Total protein)	g/dL	Biuret
ALB (Albumin)	g/dL	BCG
BUN (Blood urea nitrogen)	mg/dL	Urease, UV
CRE (Creatinine)	mg/dL	Modified Jaffe
CHO (Total Cholesterol)	mg/dL	CHOD
TG (Triglyceride)	mg/dL	Enzymatic-Trinder
HDLC (High-density lipoproteins)	mg/dL	Direct method
LDLC (Low-density lipoproteins)	mg/dL	Direct method
GLU (Glucose)	mg/dL	GOD-POD
IP (Inorganic phosphorus)	mg/dL	Molybdate
Mg (Magnesium)	mg/dL	Xylidyl blue
Ca (Calcium)	mg/dL	Arsenazo III
Fe (Iron)	ug/dL	Ferenc S
Na^+ (Sodium)	mmol/L	Direct ISE
K^+ (Potassium)	mmol/L	Direct ISE
Cl^- (Cloride)	mmol/L	Direct ISE
CK (Creatine Kinase)	U/L	IFCC
CK-MB (Creatine Kinase MB)	U/L	Immunoinhibition, NAC activated
CRP (C-reactive protein)	mg/L	Immunoturbidimetry

(8) Histopathological analysis

점 수	0	1	2	3	4
Inflammation (%)	None	<25	25–50	51–75	>75
Intimal Proliferation (%)	None	<25	25–50	51–75	>75
Endothelial loss (%)	None	<25	25–50	51–75	>75
Thrombosis	None	Minimal, Focal	Mild, Muitifocal	Moderate, Regionally diffuse	Severe, marked diffuse or total luminal occlusion
Hemorrhage	None	Focal, Occasional	Multifocal, Regional	Regionally diffuse	100 % RBC
Medial injury	None	Focal disruption of IEL	Widespread disruption of the IEL	Medical tear not extending beyond the EEL	Medical tear with involvement of the EEL

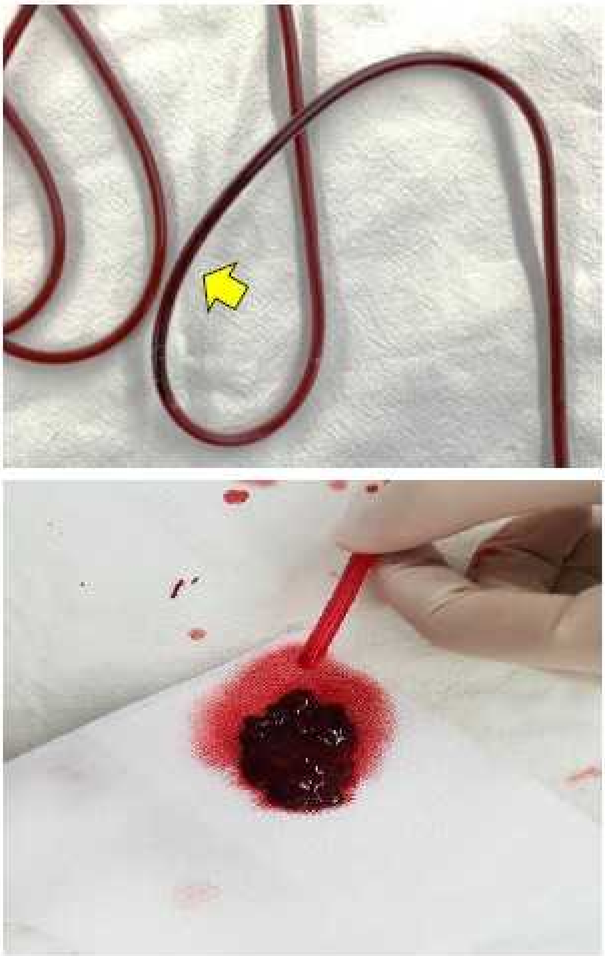

Figure 1. Production of autologous blood clot using beagle dogs.

Histopathological staining results

조직병리 염색 결과

신청자	성명	김종민
	기관명	오송첨단의료산업진흥재단 실험동물센터
	과제명	혈류_스텐트
염색항목		H&E stain, MT stain
검체채취일		2019.08.26
접수번호		190826D
시험종료 후 검체 처리 방법		습장기 (보관/1년), 파라핀블록 (보관/1년), 조직 슬라이드 (보관/1년)
동물종		Beagle, male

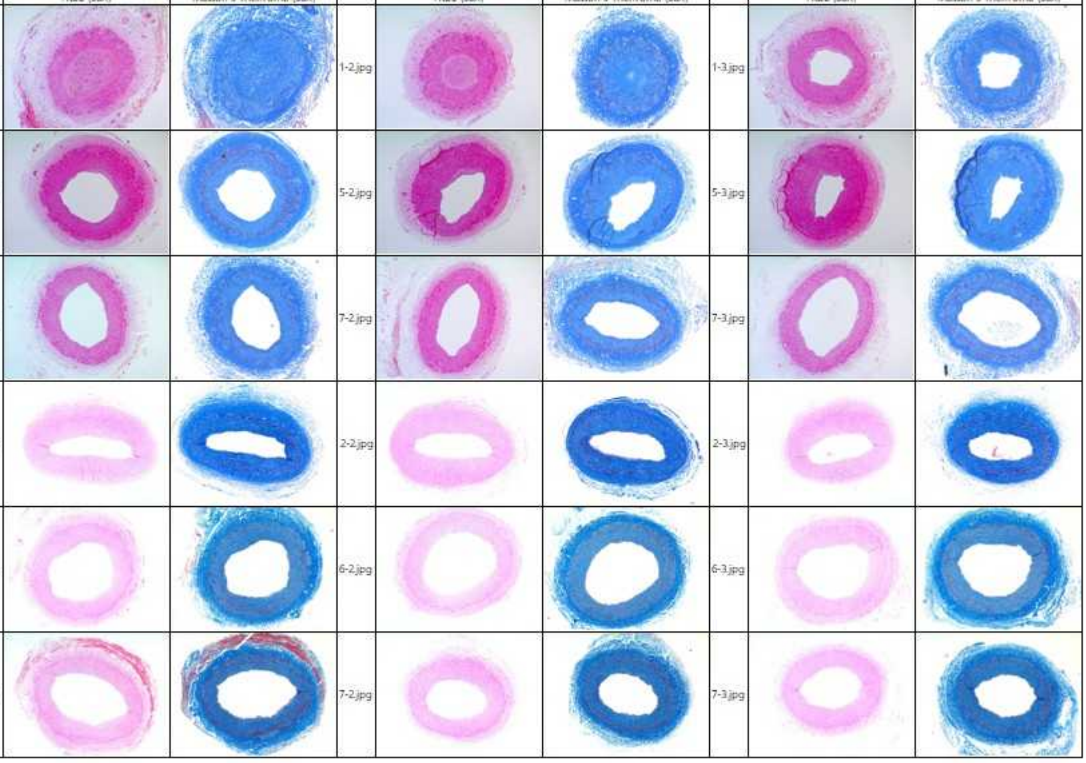

		Distal			Middle			Proximal	
		H&E (50X)	Masson's Trichrome (50X)		H&E (50X)	Masson's Trichrome (50X)		H&E (50X)	Masson's Trichrome (50X)
#1 : Solitaire control (Rt OA)	1-1.jpg			1-2.jpg			1-3.jpg		
#5 : Solitaire control (Rt OA)	5-1.jpg			5-2.jpg			5-3.jpg		
#7 : Solitaire control (Rt OA)	7-1.jpg			7-2.jpg			7-3.jpg		
#9 : Solitaire control (Rt OA)	2-1.jpg			2-2.jpg			2-3.jpg		
#11 Solitaire control (Rt OA)	6-1.jpg			6-2.jpg			6-3.jpg		
#13 Solitaire control (Rt STA)	7-1.jpg			7-2.jpg			7-3.jpg		

KBIOHEALTH

Histopathological staining results

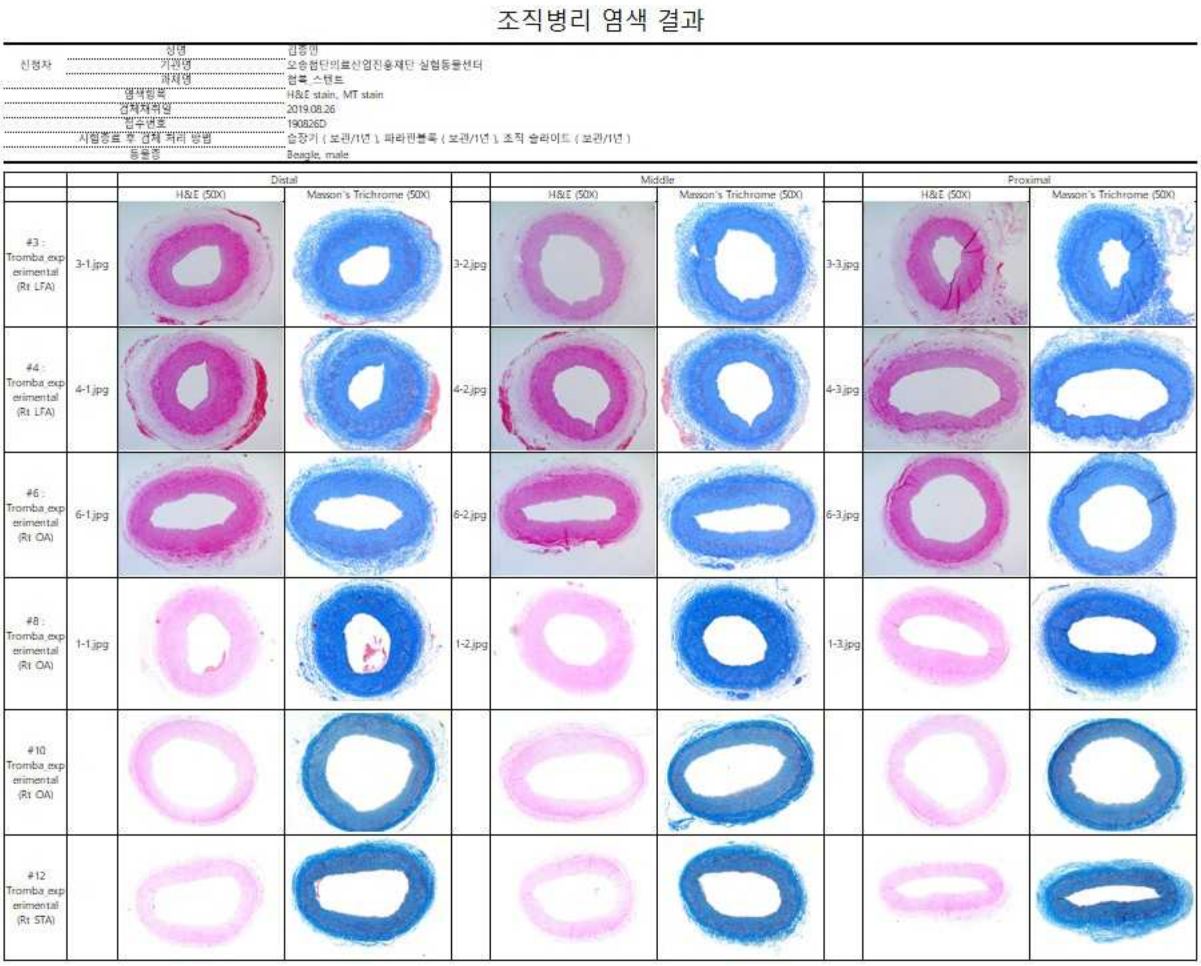

조직병리 염색 결과

신청자	성명	[illegible]
	기관명	오송첨단의료산업진흥재단 실험동물센터
	과제명	[illegible]_스텐트
염색항목		H&E stain, MT stain
검체채취일		2019.08.26
접수번호		190826D
시험종료 후 검체 처리 방법		습장기 (보관/1년), 파라핀블록 (보관/1년), 조직 슬라이드 (보관/1년)
동물종		Beagle, male

		Distal			Middle			Proximal	
		H&E (50X)	Masson's Trichrome (50X)		H&E (50X)	Masson's Trichrome (50X)		H&E (50X)	Masson's Trichrome (50X)
#3 : Tromba_experimental (Rt LFA)	3-1.jpg			3-2.jpg			3-3.jpg		
#4 : Tromba_experimental (Rt LFA)	4-1.jpg			4-2.jpg			4-3.jpg		
#6 : Tromba_experimental (Rt OA)	6-1.jpg			6-2.jpg			6-3.jpg		
#8 : Tromba_experimental (Rt OA)	1-1.jpg			1-2.jpg			1-3.jpg		
#10 Tromba_experimental (Rt OA)									
#12 Tromba_experimental (Rt STA)									

Korean Original version

최종보고서

허혈성 뇌졸중 치료를 위한 스텐트 리트리버의 안전성 및 유효성 확인

시 험 번 호 : 19-051-DE

시험책임자 : 김 상 우

시 험 기 간 : 2019.01.01 ~ 2019.12.31

시험의뢰자 : ㈜시지바이오

의뢰책임자 : 김 성 현

오송첨단의료산업진흥재단 실험동물센터
OSONG MEDICAL INNOVATION FOUNDATION LABORATORY ANIMAL CENTER

충청북도 청주시 흥덕구 오송읍 오송생명로 123번지 Tel.043)200-9840, Fax.043)200-9805

시험책임자 진술서

시험제목 : 허혈성 뇌졸중 치료를 위한 스텐트 리트리버의 안전성 및 유효성 확인

본 시험은 오송첨단의료산업진흥재단 동물실험운영위원회에서 승인 (KBIO-IACUC-2019-051) 받은 내용에 따라 실시하였으며, 그 외 시험 결과에 영향을 미칠 수 있을 만한 요인은 확인되지 않았다.

2020 년 01 월 21 일

실험책임자 : 김 상 [illegible]
소 속 : 오송첨단의료산업진흥재단 실험동물센터
주 소 : 충청북도 청주시 흥덕구 오송읍 오송생명로 123번지
연락처 : ☎ 043-200-9851, 043-200-9805 (Fax)
E-mail : swkim@kbiohealth.kr

시험개요

**시험제목**	허혈성 뇌졸중 치료를 위한 스텐트 리트리버의 안전성 및 유효성 확인
**시험목적**	비글견에서 자가혈전을 제작하여 혈관 폐색 동물 모델을 구축한 후 스텐트 리트리버 시술을 통한 혈관 개통성을 확인하였다. 또한, 임상병리학적 분석을 실시하여 혈액학 및 혈액생화학 검사상의 지표를 확인하고 조직병리학적 분석을 실시하여 혈관 내피 손상 및 염증 유발 정도 등을 확인하였다.
**시험의뢰자**	명 칭 : ㈜시지바이오<br>소 재 지 : 경기도 성남시 사기막골로 52 선텍시티 2차 111호<br>의뢰책임자 : 김성현<br>연 락 처 : ☎ 031-748-8211 (Fax) 031-748-8222
**시험기관**	명 칭 : 오송첨단의료산업진흥재단 실험동물센터<br>소 재 지 : 충청북도 청주시 흥덕구 오송읍 오송생명로 123<br>시험책임자 : 김상우<br>연 락 처 : ☎ 043-200-9851 (Fax) 043-200-9805

시험일정	2019 년 05 월 12 일 : 동물시험계획서 승인
	2019 년 06 월 12 일 : 실험동물 반입
	2019 년 06 월 19 일 : 실험동물 순화 종료
	2019 년 06 월 20 일 : 자가혈전 제작 및 스텐트 리트리버 시술 (#1)
	2019 년 06 월 28 일 : 자가혈전 제작 및 스텐트 리트리버 시술 (#2~3)
	2019 년 07 월 11 일 : 자가혈전 제작 및 스텐트 리트리버 시술 (#4)
	2019 년 07 월 19 일 : 자가혈전 제작 및 스텐트 리트리버 시술 (#5~6)
	2019 년 07 월 22 일 : 시술 1개월 후 혈관 개통성 재확인, 부검 (#1)
	2019 년 07 월 29 일 : 시술 1개월 후 혈관 개통성 재확인, 부검 (#2~3)
	2019 년 08 월 09 일 : 시술 1개월 후 혈관 개통성 재확인, 부검 (#4)
	2019 년 08 월 16 일 : 시술 1개월 후 혈관 개통성 재확인, 부검 (#5~6)
	2019 년 08 월 23 일 : 자가혈전 제작 및 스텐트 리트리버 시술 (#7~8)
	2019 년 09 월 20 일 : 자가혈전 제작 및 스텐트 리트리버 시술 (#9~10)
	2019 년 09 월 23 일 : 시술 1개월 후 혈관 개통성 재확인, 부검 (#7~8)
	2019 년 09 월 26 일 : 자가혈전 제작 및 스텐트 리트리버 시술 (#11~12)
	2019 년 10 월 23 일 : 시술 1개월 후 혈관 개통성 재확인, 부검 (#9~12)
	2019 년 11 월 04 일 : 조직병리학적 분석 실시
	2019 년 12 월 02 일 : 조직병리학적 판독 실시
	2020 년 01 월 17 일 : 최종보고서(안) 제출
	2020 년 01 월 21 일 : 최종보고서 제출

주요시험관계자

분 야	성 명	담 당 업 무
사육관리	김 종 현, DVM, Ph.D.	동물사육책임자
동물시험	김 상 우, DVM, MS.	동물시험책임자
	유 래 형, DVM, Ph.D.	동물시험담당자
	윤 지 영, MS.	동물시험담당자
	문 형 호, MS.	동물시험담당자
	김 종 민, MS.	동물시험담당자
	김 성 훈, MS.	동물시험담당자
	이 선 원, MS.	동물시험담당자
자가혈전 제작	김 종 민, MS.	동물시험담당자
	김 성 훈, MS	동물시험담당자
스텐트 리트리버 시술	이 상 헌, MD, Ph.D. [a]	시술수행책임자
	김 상 우, DVM, MS.	동물시험책임자
혈관 개통성 확인	이 상 헌, MD, Ph.D. [a]	시술수행책임자
	김 종 민, MS.	동물시험담당자
임상병리 분석	김 진 아, MS.	임상병리담당자
조직병리 분석	최 지 현, MS.	조직병리담당자
조직병리 판독	손 우 찬, DVM, Ph.D. [b]	조직병리판독자

a) 신경과, 고려대학교 안산병원 (경기도 안산시 단원구 적금로 123)

b) 실험동물병리검사실, 서울아산병원 (서울특별시 송파구 올림픽로 43길 88)

1. 요 약

본 시험은 비글견 12마리를 이용하여 자가혈전을 제작하고 혈관 폐색을 유도한 후 대조기기 (명칭: Solitaire™ FR) 혹은 시험기기 (명칭: 중심순환계 색전제거용 카테터) 스텐트 리트리버를 군 구성에 따라 각각 시술하였다. 혈관조영술 (Angiography)을 이용하여 폐색된 혈관의 개통성을 확인하였고, 임상병리학적 분석을 실시하여 혈액학 및 혈액생화학 검사상의 지표를 비교 평가하였다. 또한, 시술 1개월 후 혈관조영술을 이용하여 혈관의 개통성을 재확인하였고, 스텐트 리트리버가 통과한 우측 경동맥을 적출하여 조직병리학적 분석을 실시하였다.

1) 혈관조영술을 실시한 결과, 자가혈전을 주입한 혈관에서 혈류의 흐름이 차단되는 것으로 보아 혈관 폐색이 안정적으로 유도되었음을 확인하였다.

2) 대조기기 (Solitaire™ FR) 및 시험기기 (중심순환계 색전제거용 카테터) 스텐트 리트리버 모두 폐색된 혈관에 문제없이 진입하여 전개되는 것을 확인하였다.

3) 혈관 개통성을 평가한 결과, 대조군과 실험군 간의 Full recanalization (자가혈전에 의한 diameter reduction이 관찰되지 않는 혈관)은 비슷한 수준으로 확인되었으며, 시술 1개월 후에도 대조군과 실험군 모두 해당 혈관이 개통을 유지하였다.

4) 임상병리학적 검사를 실시한 결과, 혈액학 및 혈액생화학 검사의 전 항목에서 대조군과 실험군 간의 유의미한 차이는 관찰되지 않았다.

5) 조직병리학적 검사를 실시한 결과, 대조군 및 실험군에서 약한 (+2) 정도의 혈관내막증식이 관찰되었다. 또한, 실험군에서는 혈관내피세포의 소실과 혈관중간막 손상이 관찰되었으나 미약한 (+1) 정도로 관찰되었다.

이상의 결과를 종합하여 볼 때, 대조기기 (Solitaire™ FR)와 시험기기 (중심순환계 색전제거용 카테터) 스텐트 리트리버 시술로 인해 폐색된 혈관이 모두 개통되는 것을 관찰하였으며, 시술로 인한 체내 염증성 반응과 심한 혈관 손상은 모두 발생하지 않는 것으로 확인하였다.

2. 재료 및 방법

1) 시험기기

(1) 명칭 : 중심순환계 색전제거용 카테터

(2) 용도 : Catheter, Thrombus Retriever

(3) 공급원 : ㈜시지바이오

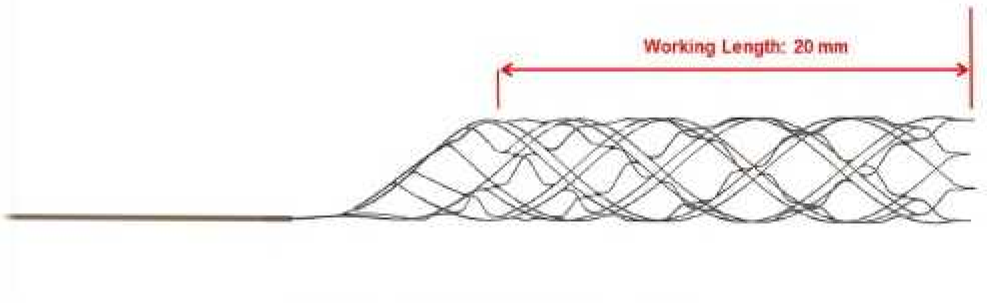

< 중심순환계 색전제거용 카테터 모형도 >

2) 대조기기

(1) 명칭 : Solitaire™ FR

(2) 용도 : Percutaneous Catheter

(3) 공급원 : ㈜시지바이오

3) 시험계

(1) 종 및 계통 : 비글견 (Beagle dog)

(2) 성별 : Male

(3) 입수시 주령 : 5 개월령

(4) 체중 : 6.0~7.7 kg

(5) 동물수 : 12마리

(6) 공급원 : Marshall BioResources / 우정BSC

(7) 시험계의 선택 이유

혈관 폐색을 유도한 후 대조기기 및 시험기기 스텐트 리트리버의 유효성을 평가하기 위해서는 비글견의 동맥 혈관크기가 가장 적합하다고 판단되었으며, 다수의 문헌에서 허혈성 뇌졸중 (스텐트 리트리버 전임상시험) 실험동물로 Canine 모델을 사용하였다 (Manik Mehra, *J NeuroIntervent*, 2012;4:307-313).

(8) 검역 및 순화

동물 입수 시 공급처에서 제공한 성적서를 참고하여 입수 동물의 검수검역을 실시하였으며, 7일간의 순화기간 중에 매일 1 회 일반증상을 관찰하여 건강한 동물만을 실험에 제공하였다.

4) 사육환경

(1) 환경조건

본 실험은 온도 23 ± 3 ℃, 상대습도 55 ± 15 %, 환기횟수 10 ~ 20 회/hr, 조명시간 12 시간 (오전 8 시 점등 ~ 오후 8 시 소등) 및 조도 150 ~ 300 Lux로 설정된 오송첨단의료산업진흥재단 실험동물센터내 개사육실에서 수행하였다. 실험자들은 모두 고압증기멸균 (121 ℃, 20 분)한 작업복과 보호장구를 착용하고 작업을 실시하였다.

(2) Cage, 사육밀도 및 사육상자의 식별

AAALAC guideline 기준 (0.74m^2) 이상 넓이의 케이지 (0.9m^2)에서 1 마리/cage로 사육하였으며, 사육케이지에 시험번호 및 동물번호를 기입한 개체식별카드를 부착하였다. Environment enrichment를 위하여 Kong classic dog toy를 제공하였다.

(3) 사료 및 물의 급여방법

시험에 공급된 사료는 방사선조사로 멸균된 시험동물용 고형사료 (2925 Telan Global 25 % Protein Dog Diet)를 급여하였으며, 사료의 성분분석 성적서를 검토한 결과, 사료조성 및 오염물질검사에서 시험에 영향을 줄만한 요인은 없었다. 물은 RO수 (역삼투증류수) 제조 및 공급장치에서 생산된 초순수를 차아염소나트륨 (NaOCl) 및 UV 장치를 거쳐 살균한 후, 자동급수 노즐을 통해 자유롭게 섭취하도록 하였으며, 공인기관 (충북보건환경연구원, 충북 청원군 오송읍 오송생명로 1로 184) 에 의뢰하여 정기적인 수질검사를 실시한 결과, '먹는물수질기준적합' 판정을 받았다.

(4) 동물실험윤리규정의 준수

본 시험은 AAALAC 인증을 받은 대한민국 보건복지부 산하 기타 공공기관, 오송첨단의료산업진흥재단 실험동물센터에서 실시하였으며, 재단 IACUC에서 승인받은 동물시험승인신청서 (KBIO-IACUC-2019-051) 내용을 준수하였다.

5) 시험군 구성

군	적용 기기	개체 수 (n)	개체 번호 (#)	시술 혈관	관찰기간 (F/U)
대조군	Solitaire™ FR	6	#1	Rt-Occipital	1 개월
			#4	Rt-Occipital	
			#6	Rt-Superficial	
			#8	Rt-Occipital	
			#10	Rt-Occipital	
			#12	Rt-Superficial	
실험군	중심순환계 색전제거용 카테터	6	#2	Rt-Linguofacial	1 개월
			#3	Rt-Linguofacial	
			#5	Rt-Occipital	
			#7	Rt-Occipital	
			#9	Rt-Occipital	
			#11	Rt-Superficial	

6) 실험방법

(1) 자가혈전 제작

가) 실험동물센터 개사육실 내 처치실에서 비글견 요측피정맥을 통해 혈액 10 ml을 채혈하였다.

나) 채혈한 혈액에 bovine thrombin 25 IU를 첨가한 후 교반하였다.

다) 교반한 혈액을 실리콘 튜브에 주입한 후 상온에서 2시간 incubation 하였다.

라) Incubation이 끝나면 4℃ 냉장 상태에서 24시간동안 보관하였다.

(2) 실험동물 마취

가) 실험동물센터 내 수술실 1번방에서 순화 사육된 비글견을 준비하였다.

나) 조레틸 (Birbac korea, 서울, 대한민국) 5 mg/kg와 럼푼 (Bayer korea, 안산, 대한민국) 2 mg/kg을 근육 주사하여 전마취를 유도하였다.

다) 전마취를 유도한 후 기도삽관을 실시하고 흡입마취기 (Fabius GS premium, Drager, 독일)에 연결시켜 isoflurane (<3 %)로 호흡마취를 유지하였다.

라) 환축 모니터링 시스템 (Vista 120, Drager, 독일)을 이용하여 동물의 마취상태 (심전도, 산소포화도, 이산화탄소 농도)를 상시 관찰하였다.

(3) 혈관 폐색모델 제작

가) 실험동물센터 내 혈관조영실에서 비글견의 대퇴동맥을 노출시킨 후 8 Fr angio sheath를 삽입하였다.

나) 6 Fr 가이딩 카테터를 이용하여 외경동맥 혈관까지 진입하였다.

다) 혈관 폐색을 유도할 적절한 내경 크기의 혈관을 선정하였다.

라) 시술 1일 전에 제작한 자가혈전을 10 mm 크기로 잘라 가이딩 카테터 내로 주입하였다.

마) 자가혈전 주입 후 15분간 embedding을 실시하였다.

바) 혈관조영 촬영기 (Artis Zee Multi-Purpose, Siemens, 독일)을 이용하여 자가혈전으로 인한 혈관 폐색을 확인하였다.

(4) 스텐트 리트리버 시술

가) 실험동물센터 내 혈관조영실에서 폐색이 유도된 혈관에 마이크로 카테터를 통과시켰다.

나) 마이크로 카테터 내로 대조기기 혹은 시험기기 스텐트 리트리버를 진입하였다.

다) 마이크로 카테터를 후퇴시키면서 스텐트 리트리버를 전개하였다.

라) 전개된 상태에서 5분간 recapture 시간을 유지하였다.

마) 마이크로 카테터와 스텐트 리트리버를 함께 회수하였다.

(5) 혈관 개통성 평가

가) 폐색된 혈관에서 스텐트 리트리버를 시술한 후 자가혈전에 의한 diameter reduction과 혈관 원위부의 재관류를 평가하였다.

나) 폐색된 혈관의 개통 정도에 따라 Full recanalization, Near complete recanalization, Partial recanalization, No recanalization으로 분류하였다.

다) 스텐트 리트리버 시술 1개월 후 혈관조영 촬영을 재실시하여 해당 혈관의 개통성을 재확인하였다.

분 류	분류방법
Full recanalization	혈류의 저류가 관찰되지 않으며 초기 혈류 상태와 비교하여 차이가 없는 완전 재관류를 의미. 또는 폐색부위 혈전에 의한 diameter reduction이 관찰되지 않는 재관류를 의미함.
Near complete recanalization	혈류의 저류가 관찰되나 초기 혈류 상태와 비교하여 차이가 없는 완전 재관류를 의미. 또는 폐색부위 혈전에 의한 diameter reduction이 20 % 미만으로 관찰되는 재관류를 의미함.
Partial recanalization	초기 혈류 상태와 비교하여 재관류를 의미. 또는 폐색부위 혈전에 의한 diameter reduction이 20 % 이상으로 관찰되는 재관류를 의미함.
No recanalization	재관류가 되지 않음을 의미함.

(6) 임상병리학적 분석 (혈액학 검사)

가) 실험동물센터 내 임상병리실에 위치한 자동혈액 분석기 (ADVIA 2120i, Siemens Healthineers, 미국)를 이용하여 각 시약에 맞는 표준물질로 정도 관리를 완료한 후 진행하였다.

나) 채혈한 혈액을 3 ml K2 EDTA 튜브 (BD Vacutainer 367856, BD, 미국)에 주입하여 혈액을 항응고시켰다.

다) 검사 시작 전까지 회전혼합기 (Lab Companion CRT-350, Jeio Tech, 대한민국)에 장착하여 전혈을 균질화 시킨 후 아래의 항목에 대하여 검사를 실시하였다.

분 류	항 목	단 위	측정방법
CBC	WBC (White blood cell count)	$10^3/\mu L$	Flowcytometry
	RBC (Red blood cell count)	$10^6/\mu L$	Flowcytometry, Isovolumetry
	HCT (Hematocrit)	%	(RBC×MCV)÷10
	HGB (Hemoglobin conc.)	g/dL	Modified CN met-Hb method
	MCV (Mean corpuscular volume)	fL	Histogram
	MCH (Mean corpuscular Hb)	Pg	(HGB÷RBC)×10
	MCHC (Mean corpusc. Hb conc.)	g/dL	[HGB÷(RBC×MCV)]×1,000
	PLT (Platelet)	$10^3/\mu L$	Flowcytometry
WBC Differential Count	%NEU (Neutrophil)	%	(NEUT x 100)÷WBC
	%LYM (Lymphocyte)	%	(LYM x 100)÷WBC
	%MONO (Monocyte)	%	(MONO x 100)÷WBC
	%EOS (Eosinophil)	%	(EOS x 100)÷WBC
	%BASA (Basophil)	%	(BASO x 100)÷WBC
	%LUC (Large unstained cells)	%	(LUC x 100)÷WBC
	#NEU (absolute NEU)	$10^3/\mu L$	Flowcytometry, Peroxidase stain
	#LYM (absolute LYM)	$10^3/\mu L$	Flowcytometry, Peroxidase stain

	#MONO (absolute MONO)	$10^3/\mu L$	Flowcytometry, Peroxidase stain
	#EOS (absolute EOS)	$10^3/\mu L$	Flowcytometry, Peroxidase stain
	#BASO (absolute BASO)	$10^3/\mu L$	Flowcytometry, Peroxidase stain
	#LUC (absolute LUC)	$10^3/\mu L$	Noise-Lymph Histogram
RET	RET (Reticulocyte)	%	RBC x (RET÷100) x 1000
	RET absolute	$10^9/\mu L$	Flowcytometry, Iso-volumetry, Absorbance(RNA content)
	ESR (Erythrocyte sedimentation rate)	mm	Westergren method

(7) 임상병리학적 분석 (혈액생화학 검사)

가) 실험동물센터 내 임상병리실에 위치한 자동혈생화학 분석기 (Konelab PRIME 60i, Thermo scientific, 핀란드)를 이용하여 각 시약에 맞는 표준물질로 정도 관리를 완료한 후 진행하였다.

나) 채혈한 혈액을 5 ml SST II advance 튜브 (BD Vacutainer 367957, BD, 미국)에 주입하고 15~20분간 상온에 방치하여 응고시켰다.

다) 응고된 혈액을 1,700 xg로 10분간 원심분리 (CF16RN, HITACHI, 일본)하여 상층의 혈청만을 분리한 후 아래의 항목에 대하여 검사를 실시하였다.

항 목	단 위	측정방법
AST (Aspartate aminotransferase)	U/L	IFCC
ALT (Alanine aminotransferase)	U/L	IFCC
ALP (Alkaline phosphatase)	U/L	SCE (DEA buffer)
GGT (Gamma-glutanyltransferase)	U/L	IFCC
LDH (Lactate dehydrogenase)	U/L	SCE
BIL (Total bilirubin)	mg/dL	Diazotized sulf. Acid
PRO (Total protein)	g/dL	Biuret
ALB (Albumin)	g/dL	BCG
BUN (Blood urea nitrogen)	mg/dL	Urease, UV
CRE (Creatinine)	mg/dL	Modified Jaffe
CHO (Total Cholesterol)	mg/dL	CHOD
TG (Triglyceride)	mg/dL	Enzymatic-Trinder
HDLC (High-density lipoproteins)	mg/dL	Direct method
LDLC (Low-density lipoproteins)	mg/dL	Direct method
GLU (Glucose)	mg/dL	GOD-POD
IP (Inorganic phosphorus)	mg/dL	Molybdate
Mg (Magnesium)	mg/dL	Xylidyl blue
Ca (Calcium)	mg/dL	Arsenazo III
Fe (Iron)	ug/dL	Ferenc S
Na^+ (Sodium)	mmol/L	Direct ISE
K^+ (Potassium)	mmol/L	Direct ISE

Cl^- (Cloride)	mmol/L	Direct ISE
CK (Creatine Kinase)	U/L	IFCC
CK-MB (Creatine Kinase MB)	U/L	Immunoinhibition, NAC activated
CRP (C-reactive protein)	mg/L	Immunoturbidimetry

(8) 조직병리학적 분석

가) 실험동물센터 내 조직병리실에서 10 % 중성 포르말린에 고정된 우측 경동맥 조직을 탈수, 투명, 침투, 포매 과정을 거쳐 파라핀 블록을 제작하였다.

나) 제작된 파라핀 블록을 4 ㎛ 두께로 삭정하여 조직 슬라이드를 제작한 후 Hematoxylin&Eosin (H&E) 염색과 Masson's trichrome (MT) 염색을 실시하였다.

다) 조직병리 판독은 서울아산병원에서 수행하였으며, Hematoxylin&Eosin (H&E) 염색이 완료된 조직 슬라이드를 통해 아래의 항목에 대하여 검사를 실시하였다.

점 수	0	1	2	3	4
Inflammation (%)	None	<25	25-50	51-75	>75
Intimal Proliferation (%)	None	<25	25-50	51-75	>75
Endothelial loss (%)	None	<25	25-50	51-75	>75
Thrombosis	None	Minimal, Focal	Mild, Multifocal	Moderate, Regionally diffuse	Severe, marked diffuse or total luminal occlusion
Hemorrhage	None	Focal, Occasional	Multifocal, Regional	Regionally diffuse	100 % RBC
Medial injury	None	Focal disruption of IEL	Widespread disruption of the IEL	Medical tear not extending beyond the EEL	Medical tear with involvement of the EEL

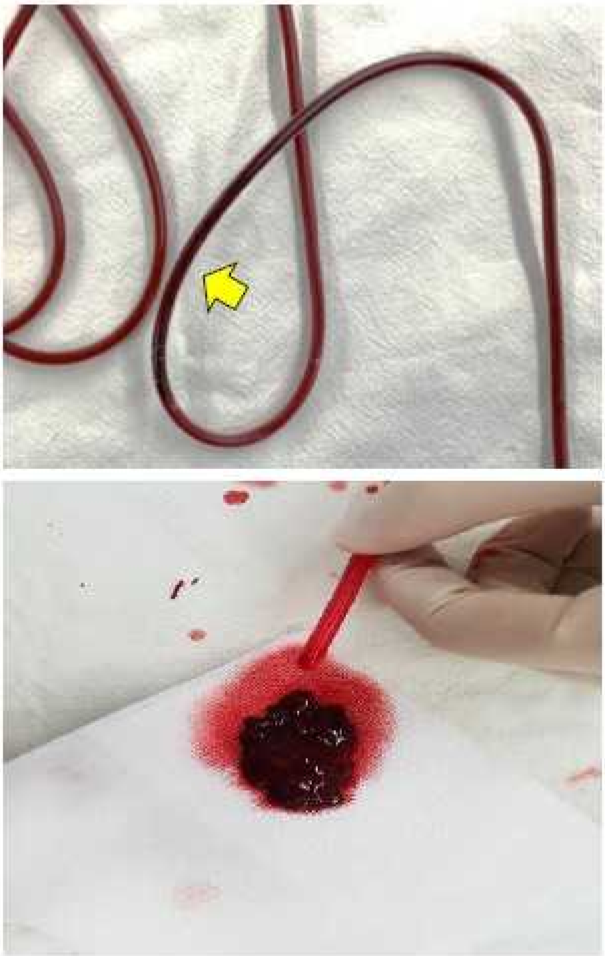

Figure 1. Production of autologous blood clot using beagle dogs.

조직병리 염색 결과

신청자	성명	김종민
	기관명	오송첨단의료산업진흥재단 실험동물센터
	과제명	첨록_스텐트
염색항목		H&E stain, MT stain
검체채취일		2019.08.26
접수번호		190826D
시험종료 후 검체 처리 방법		습장기 (보관/1년), 파라핀블록 (보관/1년), 조직 슬라이드 (보관/1년)
동물종		Beagle, male

		Distal			Middle			Proximal	
		H&E (50X)	Masson's Trichrome (50X)		H&E (50X)	Masson's Trichrome (50X)		H&E (50X)	Masson's Trichrome (50X)
#1 : Solitaire_control (Rt OA)	1-1.jpg	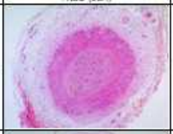	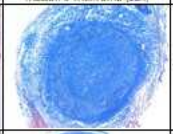	1-2.jpg	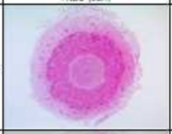	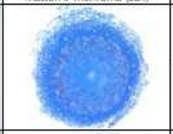	1-3.jpg	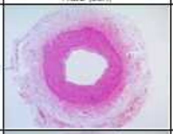	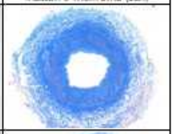
#5 : Solitaire_control (Rt OA)	5-1.jpg	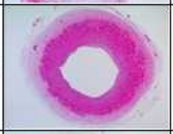	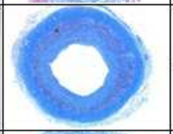	5-2.jpg	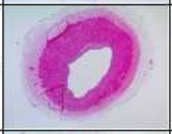	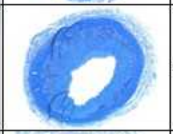	5-3.jpg	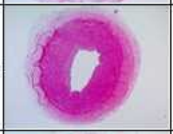	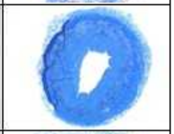
#7 : Solitaire_control (Rt OA)	7-1.jpg	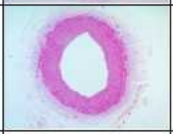	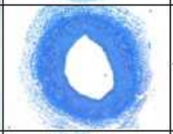	7-2.jpg	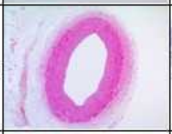	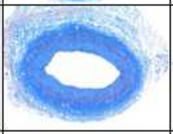	7-3.jpg	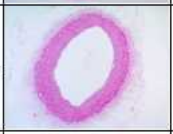	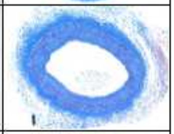
#9 : Solitaire_control (Rt OA)	2-1.jpg	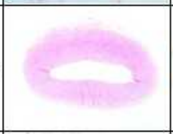	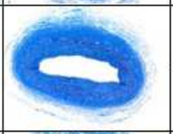	2-2.jpg	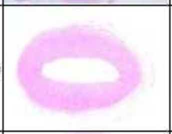	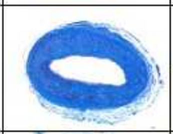	2-3.jpg	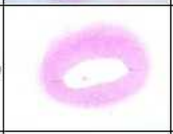	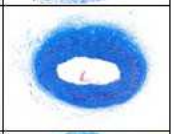
#11 Solitaire_control (Rt OA)	6-1.jpg	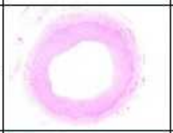	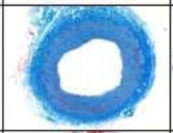	6-2.jpg	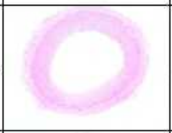	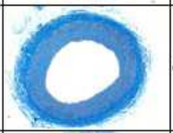	6-3.jpg	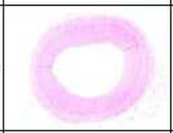	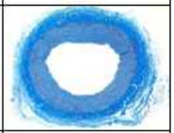
#13 Solitaire_control (Rt STA)	7-1.jpg	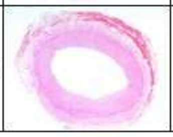	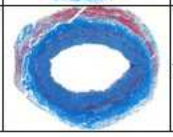	7-2.jpg	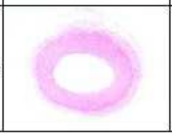	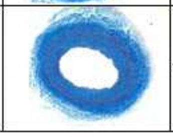	7-3.jpg	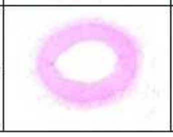	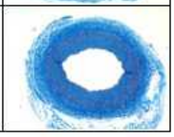

조직병리 염색 결과

신청자	성명	김종만
	기관명	오송첨단의료산업진흥재단 실험동물센터
	과제명	혈류_스텐트
염색항목		H&E stain, MT stain
검체채취일		2019.08.26
접수번호		190826D
시험종료 후 검체 처리 방법		습장기 (보관/1년), 파라핀블록 (보관/1년), 조직 슬라이드 (보관/1년)
동물종		Beagle, male

		Distal			Middle			Proximal	
		H&E (50X)	Masson's Trichrome (50X)		H&E (50X)	Masson's Trichrome (50X)		H&E (50X)	Masson's Trichrome (50X)
#3 : Tromba_experimental (Rt LFA)	3-1.jpg	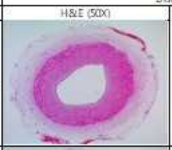	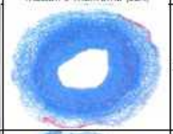	3-2.jpg	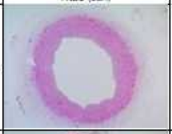	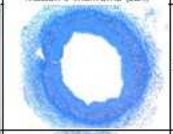	3-3.jpg	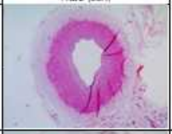	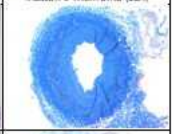
#4 : Tromba_experimental (Rt LFA)	4-1.jpg	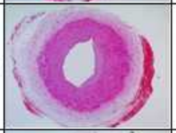	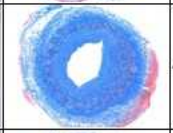	4-2.jpg	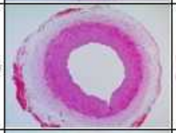	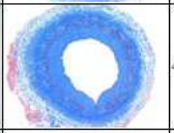	4-3.jpg	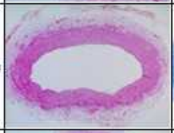	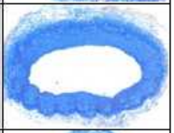
#6 : Tromba_experimental (Rt OA)	6-1.jpg	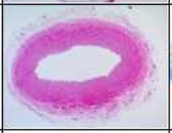	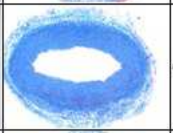	6-2.jpg	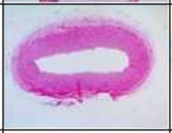	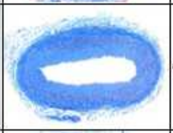	6-3.jpg	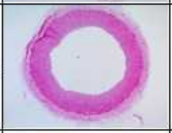	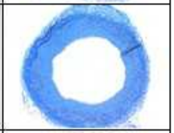
#8 : Tromba_experimental (Rt OA)	1-1.jpg	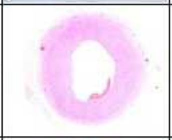	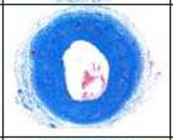	1-2.jpg	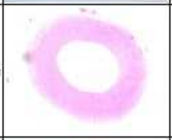	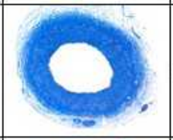	1-3.jpg	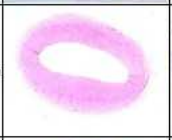	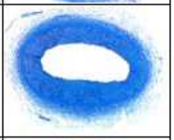
#10 Tromba_experimental (Rt OA)		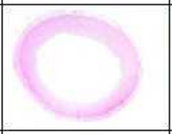	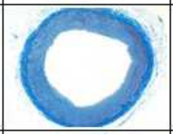		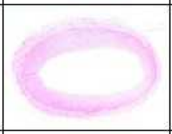	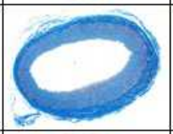		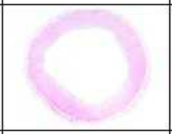	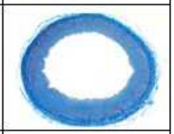
#12 Tromba_experimental (Rt STA)		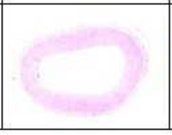	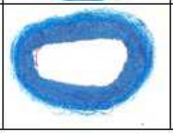		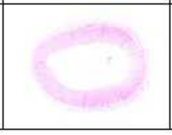	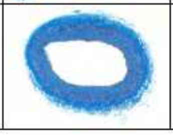		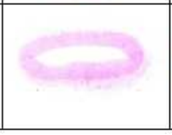	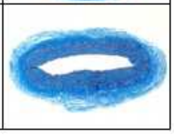
