# Supplementary material for: In vivo evaluation of histopathologic findings of vascular damage after mechanical thrombectomy with the Tromba device in a canine model of cerebral infarction
Source: PLoS One. 2022 Oct 14;17(10):e0276108. doi: 10.1371/journal.pone.0276108 (PMC9565453; doi:10.1371/journal.pone.0276108)
Supplement: Attachment — Submitted filename: Response to Reviewers.docx [file pone.0276108.s003.docx]

Thank you for the opportunity to revise our manuscript. We appreciate your careful review and constructive proposals. We believe that the manuscript has been substantially improved by following your suggestions. We have provided point-by-point responses to the reviewer’s comments, as noted below. The revisions have been developed in consultation with all coauthors, and each author has approved the final version. However, it seems that comments 1 to 13, including "Main text 1” in which you wrote: “Lesion location was defined according to SYNTAX score. But actually …" do not correspond to this manuscript. It is considered that these may be responses to other papers that have accidentally been inserted. Please kindly verify this.

Thank you for your assistance.

**Reviewer 1**

**Comments to the Author**

**Abstract: 1.**

**It is unclear from the title, as well as from the abstract, for which vessels the device is developed. Is that a thrombectomy device for coronaries? For central nervous system? For pulmonary? For deep veins?**

♦ Authors’ response

Thank you for commenting on this important point.

We have developed a thrombectomy device for the central nervous system. To be more specific, it is a device to clear the occlusion of an intra- or extra-cranial artery that caused a cerebral infarction.

The above is indicated in the title and abstract.

♦ Changes

(Page 1, Line numbers 1-2) Title

“In vivo evaluation of histopathologic findings of vascular damage after mechanical thrombectomy with the Tromba device in a canine model of cerebral infarction.”

(Page 2, Line numbers 22)

“A novel stent retriever device for in vivo mechanical thrombectomy for acute cerebral infarction has been developed.”

(Page 2, Line numbers 27)

“Through this experimental evaluation, we aimed to assess the safety and efficacy of the newly developed thrombus removal device for cerebral infarction.”

**Abstract: 2.**

**It has to be declared in the abstract, at what timepoint the animal models were sacrificed and analyzed. Considering that endothelial damage has the potential for instantaneous risk of further thrombus formation, therefore acute result is a critical aspect. On the other and it might heal over time, losing its relevance. But this would be nice to demonstrate. Since authors mention ‘intimal proliferation’, one would assume that chronic results are presented, however it is still unclear.**

♦ Authors’ response

We agree with the reviewer’s comment. To rectify this, we have added the requested information (the time point of sacrifice and analysis) in the abstract.

♦ Changes

(Page 2, Line numbers 31-33)

Angiographic and histopathologic evaluations were performed 1 month after the blood vessels underwent mechanical thrombectomy.

**Abstract: 3.**

**‘Moderate-’ and ‘slight narrowing’ are quite subjective phrasing for someone who is not fully familiar with the Grandin scale and therefore difficult to interpret for comparison. Please quantify it.**

♦ Authors’ response

We appreciate your comment. The grade of reperfusion patency was defined according to the scale introduced by Grandin et al., as follows: no narrowing, slight narrowing (25% reduction in the lumen diameter), moderate narrowing (25%–50% stenosis or 50%–75% stenosis affecting only a short segment of the vessel), and severe narrowing (50%–75% stenosis affecting a long segment of the vessel or stenosis > 75%). We have added the abovementioned quantified information in the grade of reperfusion patency of the abstract.

♦ Changes

(Page 2, Line numbers 34-40)

In the experimental group, the reperfusion patency was classified as “no narrowing” in five cases and “moderate narrowing (25%–50% stenosis)” in one case. In the control group, the reperfusion patency was classified as “no narrowing” in four cases, “moderate narrowing (25%–50% stenosis)” in one case, and “slight narrowing (less than 25% stenosis)” in one case.

**Main text:1.**

**It would be important to define, why the Solitaire FR was selected as reference. Is that the most widely used and accepted device of the field?**

♦ Authors’ response

The Solitaire FR Revascularization Device (ev3, Irvine, California) is the first dedicated blood flow restoration and thrombectomy dedicated device for the treatment of acute stroke. [Mordasini P et al, Am J Neuroradiol. 2010;31(5):972-8]

A recent pivotal study investigated whether mechanical thrombectomy could accelerate recanalization and increase the rate of revascularization. In most studies, the Solitaire FR Revascularization Device was most commonly used and these studies demonstrated rapid blood flow recovery and improvement of functional outcome after acute ischemic stroke.

♦ Changes

(Page 4, Line numbers 51-57)

“Several recent pivotal studies have investigated whether mechanical thrombectomy can accelerate recanalization and improve stroke prognosis using a self-expanding stent retriever such as the Solitaire FR Revascularization Device. These studies have demonstrated that mechanical thrombectomy leads to rapid blood flow recovery and improvement of functional outcome in acute ischemic stroke patients [1-6].”

**Main text:** **2.**

**From main text it is clear that results were ‘only’ at intermediate term after 30-days assessed. Since acute injury could have been assessed by intravascular imaging modalities, I believe the lack of acute data is a major limitation and it has to be acknowledged.**

♦ Authors’ response

We fully agree with the reviewer's comments.

In this study, imaging follow-up was performed immediately after mechanical thrombectomy and 1 month after mechanical thrombectomy, without regular follow-up investigations in between. Therefore, angiographic evaluation was not performed during the condition of acute phase vascular damage and blood flow, which is a limitation. This was noted in the limitations section.

♦ Changes

(Page 15, Line numbers 269-272)

“Third, imaging follow-up was performed immediately after mechanical thrombectomy and 1 month later, without regular follow-up investigations in between. Therefore, angiographic evaluation was not performed during the condition of acute phase vascular damage and blood flow.”

**Main text: 3.**

**In the histopathologic result section the Authors write about swine model instead of canine. Please clarify.**

♦ Authors’ response

I apologize for this typographical error. We have changed “swines” to "canines."

**Main text: 4.**

**In the limitation section Authors state: “However, histologic comparisons involving a large number of animals have been conducted in other canine models, and the results were considered to be of sufficient value.” It is unclear, which study and what device they refer to. It should be at least referenced or even described in a bit more details.**

♦ Authors’ response

We intended to conduct a study on relatively more canine models than other studies that had performed histological verifications through animal experiments. For the studies we referenced, Gralla J et al. conducted a study with 8 swines, and Mordasini et al. conducted a study with 5 swines. Brooks et al. studied 6 canines, and Nogureira et al. performed a biopsy with 2 swines and one canine. The following journals are attached as references. [11, 14, 16, 21-23]

1. [11] Gralla J et al. A dedicated animal model for mechanical thrombectomy in acute stroke. Am J Neuroradiol. 2006;27(6):1357-61

2. [14] Gralla J et al. Mechanical thrombectomy for acute ischemic stroke - Thrombus-device interaction, efficiency, and complications in vivo. Stroke. 2006;37(12):3019-24

3. [16] Nogueira RG et al. The Trevo device: preclinical data of a novel stroke thrombectomy device in two different animal models of arterial thrombo-occlusive disease. J Neurointerv Surg. 2012;4(4):295-300

4. [21] Mordasini P et al. In Vivo Evaluation of the First Dedicated Combined Flow-Restoration and Mechanical Thrombectomy Device in a Swine Model of Acute Vessel Occlusion. Am J Neuroradiol. 2011;32(2):294-300

5. [22] Brooks OW et al. A canine model of mechanical thrombectomy in stroke. J Neurointerv Surg. 2019;11(12):1243-8

6. [23] Mordasini P et al. In Vivo Evaluation of the Phenox CRC Mechanical Thrombectomy Device in a Swine Model of Acute Vessel Occlusion. Am J Neuroradiol. 2010;31(5):972-8.

♦ Changes

(Page 15, Line numbers 266-267)

However, histologic comparisons involving a large number of animals have been conducted in other studies using swine or canine models (usually less than 10 animals).[reference]

**Main text: 5.**

**A certain structural / mechanical / design comparison of the novel versus the traditional device, focusing on potential advantages, would be important for better understanding.**

♦ Authors’ response

I agree with your comment. Structural, mechanical, and design advantages and disadvantages were compared by comparing the stent structure developed by us, "the hybrid structure with open and closed cells", with "the closed cell" structure of the traditional device including the Solitaire stent. The content has been inserted into the discussion section and Figure 1B has been added to help readers understand the above.

♦ Changes

(Page 14, Line numbers 246-257)

“The thrombus removal part of the existing thrombus retrievable stent developed so far, including Solitaire, is in the form of a closed cell structure in which all cells are connected by a link to fix and recover the thrombus.. The stent of the "closed cell" structure fixes the thrombus in a relatively stable manner, so that the captured thrombus is smoothly recovered. However, due to its low flexibility and expansion power, it is difficult for this stent to adapt to the tortuous cerebral blood vessels, making it difficult to remove a thrombus in a severely bent region. This is a limitation as repeated thrombus removals can damage blood vessel walls. The hybrid cell structure is judged to be ideal because it has both the characteristics of an open cell, i.e., it can adapt well to complex and tortuous cerebral blood vessels, and a closed cell, i.e., it can stably remove and recover blood clots. In addition, a structure with a weak radial force and a structure that cannot be recaptured are the biggest disadvantages of an open cell. These can be overcome with a hybrid cell structure.”

**Reviewer #2**

**Solid data presentation with regard to this new technology**

**Comments:**

**-With the assumption that acute stroke should be acute emboli acute thrombotic clots were injected; seems reasonable. But in reality also chronic clots could migrate to the cerebral arteries, eg patient has AF. Are there any data if this technology also works in chronic clots??**

♦ Authors’ response

I understand the reviewer's question. A recent pivotal study investigated whether a mechanical thrombectomy could accelerate the potency and increase the rate of recanalization. The 5 trials are MR CLEAN, EXTEND-IA, ESCAPE, REVASCAT, and SWIFT PRIME, all published in the "New England Journal of Medicine". All these trials were performed in a similar way, and the group that underwent mechanical thrombectomy using a retrievable stent was compared with the group that did not. In most studies, mechanical thrombectomy with a retrievable stent showed rapid blood flow recovery and improved functional outcome in acute ischemic stroke. Furthermore, in most of the studies, a large number of participating patients had cardiac embolic infarction (including AF) leading to vascular occlusion due to chronic clots.

In MR CLEAN, atrial fibrillation accounted for 28.3% and 25.8% of patients in the intervention and control groups, respectively, in EXTEND-I, 31% and 34%; ESCAPE, 37% and 40%; REVASCAT, 34% and 35.9%; and SWIFT PRIME, 39% and 36%, respectively. More than 30% of these patients had cardiac embolic infarction associated with AF, which can be represented by chronic clots, demonstrating that mechanical thrombectomy is effective in thrombus removal in this patient group as well. Therefore, data relating to this technique shows that it will be effective in chronic blood clots.

The referenced papers are listed below.

-Reference-

1. Berkhemer OA, et al. A randomized trial of intraarterial treatment for acute ischemic stroke. New Engl J Med. 2015;372(1)

2. Campbell BCV, et al. Endovascular Therapy for ischemic stroke with perfusion-imaging selection. New Engl J Med. 2015;372(11):1009-18

3. Goyal M, et al. Randomized assessment of rapid endovascular treatment of ischemic stroke. New Engl J Med. 2015;372(11):1019-30

4. Jovin TG, et al. Thrombectomy within 8 hours after symptom onset in ischemic stroke. New Engl J Med. 2015;372(24):2296-306

5. Saver JL, et al. Stent-retriever thrombectomy after intravenous t-PA vs. t-PA alone in stroke. New Engl J Med. 2015;372(24):2285-95

**-The authors state throughout the manuscript that they used 12 canine models (beagles), but in the section histopathological findings they state that they used 12 swines?? This need to be clarified**

♦ Authors’ response

I apologize for this typographical error. We have changed “swines” to "canines."
