# Supplementary material for: In vivo evaluation of histopathologic findings of vascular damage after mechanical thrombectomy with the Tromba device in a canine model of cerebral infarction
Source: PLoS One. 2022 Oct 14;17(10):e0276108. doi: 10.1371/journal.pone.0276108 (PMC9565453; doi:10.1371/journal.pone.0276108)
Supplement: Attachment — Submitted filename: Response_to_Reviewers.docx [file pone.0276108.s004.docx]

Thank you for your opportunity to revise our manuscript. We appreciate your careful review and constructive suggestions. We believe that the manuscript has been substantially improved following your suggestions. We have provided point-by-point responses to the reviewers’ comments below. The revisions have been performed in consultation with all co-authors, and each author has approved the final version.

Thank you for your assistance.

**Reviewer Comments to the Author**

**1) In line with the PLOS data policy, please provide the raw histopathology images as supporting information files. Please also confirm in your response to reviewers document that all content in the supporting information files may be published under a CC-BY license, i.e. that there are no confidentiality restrictions.**

♦ Authors’ response

Raw histopathological images were additionally submitted as supporting information files. We confirmed that all content in the supporting information files may be published under a CC-BY license.

**2) Outline the rationale for using a canine model in your introduction or methods section.**

♦ Authors’ response

We have added the requested information to the Methods section.

♦ Changes

(Revised Manuscript with Track Changes - Page 5, Line numbers 94-98)

Canine models have several advantages. The size and extent of vasospasm occurring in canines are more similar to those in humans than in porcine and rodent models, allowing for more targeted endovascular treatment and the branches of the external carotid artery in the canine model have a vessel size similar to that of the human middle cerebral artery, which is sufficient for the study of novel endovascular devices for human cerebral blood vessels [11].

[11]. Mehra M, Henninger N, Hirsch JA, Chueh J, Wakhloo AK, Gounis MJ. Preclinical acute ischemic stroke modeling. Journal of Neurointerventional Surgery 2012;4:307-313.

**3) Include the following information in the methods section of the main text of the manuscript (some of this is currently detailed in the SI files, but it should be moved to the main text):**

**- The source of the animals.**

**- Details of housing, husbandry, and care, including diet, enrichment, and exercise as applicable.**

**- Animal health monitoring, including frequency and criteria and any efforts made to reduce suffering and distress, such as administering analgesics.**

**- Whether humane endpoints were in place during the study and how they were applied**

**- The methods of anesthesia and euthanasia.**

**- Any mortality that occurred outside of planned euthanasia or humane endpoints.**

**In particular, please include a description of the euthanasia and vessel retrieval before histopathology, and a description of the care of the beagles post-surgery after in effect having an induced stroke.**

♦ Authors’ response

We fully agree with the reviewer’s comment. The content has been inserted into the “Animal care and process of in vivo study” section of the Methods.

♦ Changes

(Revised Manuscript with Track Changes - Page 5, Line numbers 90-132)

To identify all experimental environments, we tested canines of the same age, weight, and size. Twelve 6-month-old canines (beagle dogs obtained from Marshall BioResources, North Rose, NY, USA) weighing between 6.0 and 8.0 kg were included in this study. Canine models have several advantages. The size and extent of vasospasm occurring in canines are more similar to those in humans than in porcine and rodent models, allowing for more targeted endovascular treatment and the branches of the external carotid artery in the canine model have a vessel size similar to that of the human middle cerebral artery, which is sufficient for the study of novel endovascular devices for human cerebral blood vessels [11]. One animal was bred in a cage (0.9 m^2^) in accordance with AAALAC guidelines (0.74 m^2^), and an individual identification card with the test and animal numbers was attached to the breeding cage. A classic dog toy was used for environmental enrichment. The conditions of the breeding environment were as follows: temperature, 23°C ± 3°C; relative humidity, 55% ± 15%; ventilation, 10–20 times/h; lighting time, 12 h (lights up at 8 am, lights out at 8 pm), and illuminance, 150–300 Lux. This study was conducted in an established canine-breeding room. For the feed supplied to the test, a solid feed for test animals (2925 Telan Global 25% Protein Dog Diet) was sterilized by irradiation. Ultrapure water produced by an RO water (reverse osmosis distilled water) production and supply device was sterilized using sodium hypochlorite and a UV device and then freely dispensed through an automatic water supply nozzle.

All experiments were conducted under general anesthesia. Preoperative anesthesia was induced by intramuscular injection of 5 mg/kg Zoletil (Bibac Korea, Seoul, Korea) and 2 mg/kg Rompun (Bayer Korea, Ansan, Korea). For postoperative pain/stress relief in experimental animals, 0.2 mg/kg of Metacam (Boehringer Ingelheim, Germany) was subcutaneously injected once daily for three days. In addition, 15 mg/kg cefazolin (Chong Kun Dang, Korea) was subcutaneously injected once daily for three days to prevent infection. For humane management of diet, exercise, and nutrition in experimental animals with stroke induced by vascular occlusion and mechanical thrombectomy, the experiment was limited to unilateral sites of the carotid artery, not to bilateral sites. During the observation period, animals treated with the stent retriever in the breeding room of the experimental animal center were visually observed for abnormal symptoms once a day, and the experimental animals had no restrictions on their daily activities, including diet.

Histopathological analysis was performed by excising vessels that had undergone mechanical thrombectomy. Anesthesia was induced by intramuscular injection of 5 mg/kg Zoletil (Bibac Korea, Seoul, Korea) and 2 mg/kg Rompun (Bayer Korea, Ansan, Korea) in the operating room of the experimental animal center. After induction of general anesthesia, airway intubation was performed, the animal was connected to an inhalation anesthesia machine (Fabius GS premium, Drager, Germany), and respiratory anesthesia was maintained with isoflurane (<3%). The animals’ anesthetic conditions (electrocardiogram, oxygen saturation, carbon dioxide concentration, etc.) were constantly monitored using a patient monitoring system (Vista 120, Drager, Germany). At the end of the experiment, 40 ml of KCl was injected intravenously after intramuscular injection to induce euthanasia, and respiration and cardiac arrest in the experimental animals were confirmed. There were no other mortalities, except planned euthanasia, and no humane endpoints in the study.
